# Assessing the reliability of Response Evaluation Criteria In Solid Tumors (RECIST): a systematic review of the factors contributing to inter-observer variability

**DOI:** 10.1186/s13244-026-02320-6

**Published:** 2026-06-30

**Authors:** Illaa Smesseim, Teresa T. Bucho, Renaud Tissier, Stevie van der Mierden, Ferry Lalezari, Jacobus A. Burgers, Stefano Trebeschi

**Affiliations:** 1 Department of Thoracic Oncology, Netherlands Cancer Institute, Amsterdam, The Netherlands; 2 Department of Pulmonary Diseases, Leiden University Medical Center, Leiden, The Netherlands; 3 Department of Radiology, Netherlands Cancer Institute, Amsterdam, The Netherlands; 4 GROW School for Oncology and Reproduction, Maastricht University, Maastricht, The Netherlands; 5 Biostatistics Unit, Netherlands Cancer Institute, Amsterdam, The Netherlands; 6 Department of Library, Netherlands Cancer Institute, Amsterdam, The Netherlands

**Keywords:** RECIST, WHO, Interobserver variability, Response evaluation, RECIST 1.1

## Abstract

**Background:**

The Response Evaluation Criteria in Solid Tumors (RECIST) have been the standard for assessing tumor response in oncology trials since 2000. Despite the use of these criteria, interobserver variability (IOV) remains a significant concern, as it can affect patient management and clinical trial outcomes. We aimed to review the current literature on factors contributing to IOV in RECIST.

**Materials and methods:**

We conducted a systematic review to summarize potential factors that can contribute to variability, variance, or reproducibility in RECIST assessments: (I) manual measurements, (II) selection of lesions, (III) 1D diameters, (IV) intra-radiologist variability, (V) the experience of the readers, (VI) local vs centralized assessment, (VII) variations of criteria used, (VIII) type of imaging, (IX) follow-up schedule. Inclusion criteria required studies to use RECIST (1.0, 1.1, or modified versions), involve multiple response evaluations and provide comparative data on the same test subjects.

**Results:**

We identified 246 studies, of which 88 met our inclusion criteria. Median sample size across studies was 50 patients (IQR: 25.8–90.8). Most studies (68.2%) used CT scans as the sole imaging modality. The most common primary tumors studied were lung and hepatocellular carcinoma (each 14.8%). Most studies showed an IOV being affected by measurement methods, lesion selection, and imaging quality. Statistical methods for IOV vary: Kappa for categorical and intraclass correlation for continuous data, with calculation differences limiting comparability and risking misleading conclusions.

**Conclusion:**

Standardized protocols are needed to study interobserver variability in RECIST and enable us to understand the current criteria and develop better ones.

**Key Points:**

- We should standardize the assessment of interobserver variability in RECIST to improve reproducibility, comparability, and clinical trial decision reliability.
- Interobserver variability in RECIST depends on measurement methods, lesion selection, and imaging quality. Inconsistent statistics hinder study comparability.
- Standardized RECIST protocols can reduce variability, improving evaluation consistency, clinical decisions, and treatment outcomes in oncology trials.

**Graphical Abstract:**

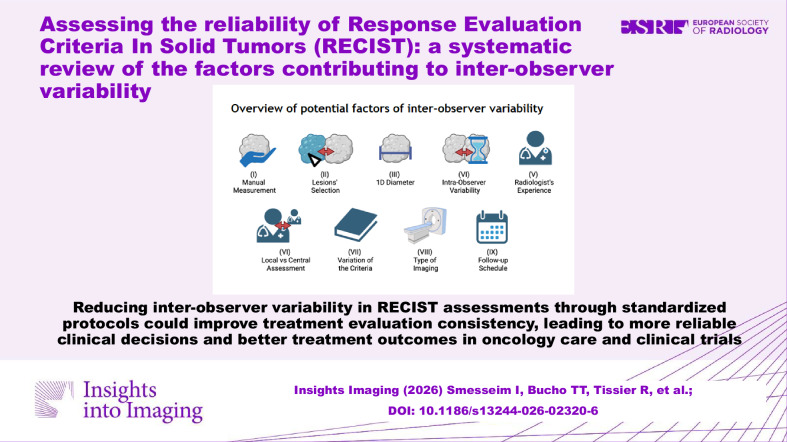

## Introduction

In the evolving landscape of oncology, the Response Evaluation Criteria In Solid Tumors (RECIST) stands as a pillar, ensuring consistency in measuring and reporting tumor responses to therapeutic interventions [1, 2]. Since its inception in 2000 [2] and its revision in 2009 [1] as RECIST 1.1, this framework has acted as the standard for evaluating therapeutic outcome in many clinical trials, effectively shaping our understanding of treatment efficacies and their impact on patient outcomes.

While RECIST has standardized tumor assessment, concerns about its reproducibility remain, mostly in the form of interobserver variability (IOV). As these inconsistencies can impact patient management and clinical trial outcomes [3, 4], it is important to understand their mechanisms, where and when these inconsistencies arise, and what can be done to mitigate or even solve them.

RECIST incorporates several standardized criteria that may help reduce interobserver variability (IOV). For example, it defines measurable lesions using minimum size thresholds and specifies cut-offs for response categorization (e.g., 30% decrease for partial response and 20% increase for progressive disease), which provide objective boundaries for classification. In addition, RECIST standardizes lesion selection and limits the number of target lesions to be measured, primarily to improve the feasibility and consistency of assessments across studies. Despite these efforts toward standardization, IOV remains a debated and persistent issue in clinical practice. Various factors can contribute to RECIST variability. Tumors may have varying appearances, in their shape and margins, for instance, which can affect how straightforward their identification and measurement is, growth patterns and therapy responses. Different imaging techniques may also depict the same tumor differently. Radiologists have unique experiences and understanding of the guidelines. All these factors lead to inconsistent interpretations and implementation of RECIST. For example, the same tumor could be classified as partially responding on one imaging technique and appear stable or progressing on another. The same tumor measured using manual diameters might be classified as stable but progressing when the measurement is repeated with a semi-automatic technique. The same tumor might be unequivocally progressing according to one radiologist, but not according to another, and so on.

A variety of studies have been conducted to quantify the influence of these various factors on IOV, but to date, there are no definitive conclusions regarding which factors influence IOV or warrant further investigation. Therefore, with this systematic review, we aim to summarize the current literature on the factors that can lead to IOV in RECIST.

## Materials and methods

### Literature search and study selection

We performed a literature search across MEDLINE (PubMed), Embase.com, and Scopus from the month of publication of the RECIST 1.1 official guidelines (December 22nd, 2008) until August 9th, 2023, searching for all publications referring to RECIST and terms of variability, variance, and reproducibility in their abstracts, titles, or keywords, and MeSH terms for PubMed. Specifically, the search terms applied for PubMed were: (“Response Evaluation Criteria in Solid Tumors”[mesh] OR RECIST[tiab] OR “Response Evaluation Criteria in Solid Tumo*”[tiab]) AND (variabilit*[tiab] OR reproducibilit*[tiab]) AND 2008/12:2024[dp]. The searches for Embase.com and Scopus can be found in Supplement 1.

This review was reported in accordance with Preferred Reporting Items for Systematic Reviews and Meta-Analyses (PRISMA) [5]. Given the substantial methodological heterogeneity in study design, imaging techniques, and outcome definitions across included studies, a formal risk-of-bias assessment using tools such as ROBINS-I was deemed not appropriate. Most studies were not designed to test interventions but to evaluate measurement reproducibility and interobserver variability, which are not easily assessed using conventional bias assessment tools. Instead, we provide a qualitative appraisal of study strengths and limitations in the “Results” and “Discussion” sections.

### Data extraction

Two independent investigators (I.S., S.T.) reviewed both titles and abstracts to assess study relevance in Rayyan, a software used for abstract screening. After the removal of duplicates and non-English articles, we identified 246 articles as potentially relevant and reviewed the full publication. Studies were considered for inclusion if they met the subsequent criteria: (1) adoption of RECIST criteria (1.0, 1.1, or modified), (2) multiple response evaluations conducted on the same test subjects (e.g., patients or phantoms) by either the same or multiple readers, and (3) a comparative analysis between evaluations executed at either the population or singular subject level. Articles such as commentaries, reviews, editorial pieces, correspondences, book sections, and subjective pieces were not considered. Duplicate results, as well as those abstracts not presented in English, and veterinary studies were likewise disregarded. Results purely based on functional imaging, including positron emission tomography (PET), functional MRI, and perfusion CT, were also excluded, as well as planar imaging, including X-ray and ultrasounds.

**Fig. 1:**
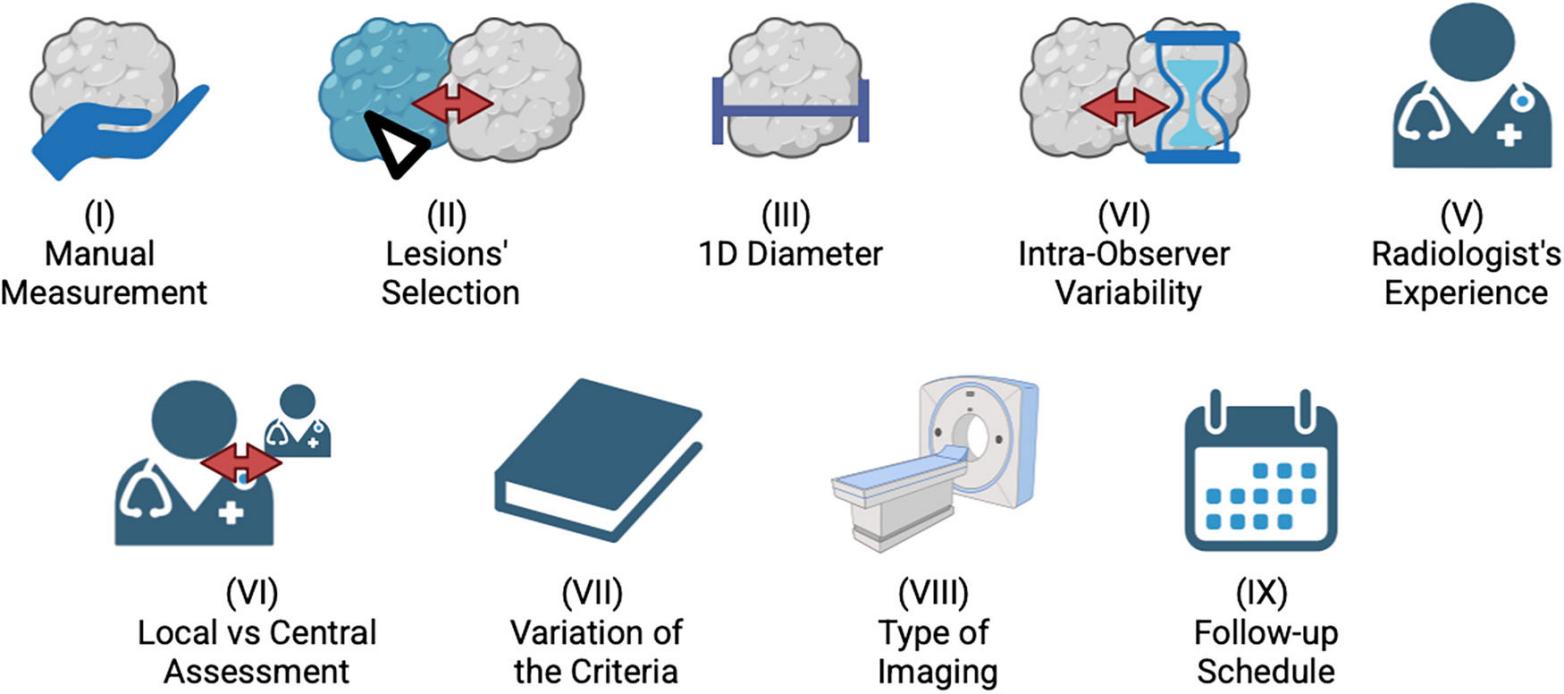
Overview of potential factors of interobserver variability

Two reviewers (I.S. and S.T.) independently extracted data from relevant studies using a standardized form containing the following questions: (1) sample size (2) data collection strategy (retrospective, prospective, or simulation/phantom), (3) imaging modality (CT, MRI or PET) (4) type of RECIST (1.0, 1.1, modified, or customized adapted by the authors, e.g., “primary tumors were excluded from the analysis”) (5) tumor type and (6) factors investigated. We identified the following potential factors contributing to IOV: (I) manual vs (semi)-automatic measurements, (II) selection of target or measurable lesions, (III) 1D vs 2D/3D diameters, (IV) intra-radiologist variability, i.e., variations in measurement repeated by the same radiologist, (V) the experience of the readers, (VI) the involvement of the radiologist in the trial, (VII) the variations of criteria used, (VIII) the type of imaging, (IX) the follow-up schedule. When full-text articles were not available online, we reached out to the corresponding authors to request the missing publications. An overview is shown in Fig. 1. General information regarding first authors, corresponding authors, year of publication, and Digital Object Identifier (DOI) was also collected.

## Results

### Study selection

**Fig. 2:**
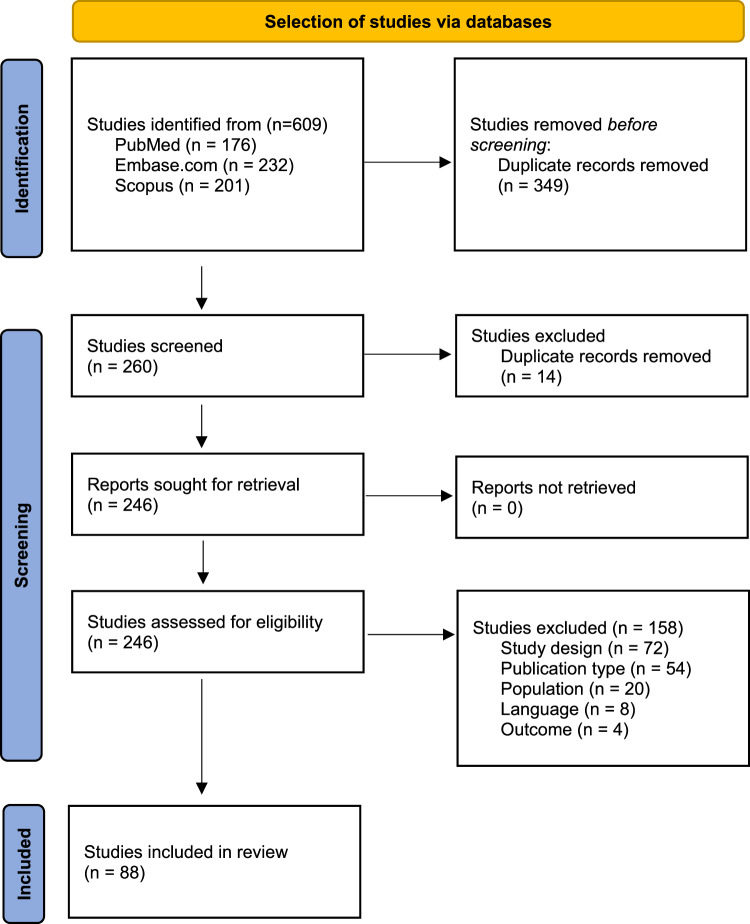
Study flowchart

The search returned *n* = 246 entries to review. *n* = 138 (56%) were excluded by both readers. Of the remaining papers, *n* = 20 (8%) papers were excluded after these were revised by consensus with a third independent reader (TTB), resulting in 88 papers, see Fig. 2.

### Sample size

The median sample size was 50.0 patients (interquartile range (IQR): 25.8–90.8). The study with the largest sample included *n* = 23,259 patients [6], and the study with the smallest sample size included three patients [7].

### Imaging modality

CT scans were used as a single modality in most studies (*n* = 60; 68.2%) [6–65], followed by MRI scans (*n* = 9; 10.2%) [66–74], and PET scans (*n* = 2; 2.3%) [75, 76]. One study used caliper or tape measurements (*n* = 1; 1.1%) [77]. The remaining studies were multi-modality: *n* = 15 (17.0%) included both CT scans and MRI scans [78–92], and one focused on CT-, MRI-, and PET scans (1.1%) [93].

### Primary tumor and study design

**Fig. 3:**
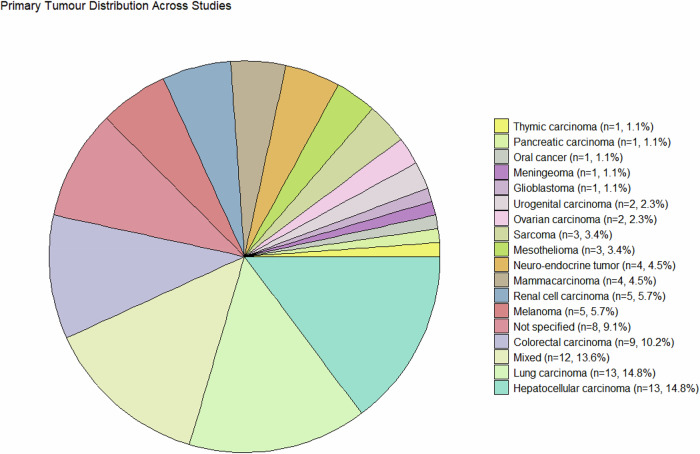
Primary tumor distribution across studies

The most common primary tumors were lung (*n* = 13; 14.8%, Fig. 3) [9, 10, 13, 22, 27, 30, 31, 36, 48, 72, 79, 88, 93], hepatocellular (*n* = 13; 14.8%) [12, 20, 33, 34, 49, 64, 71, 76, 80, 83, 84, 86, 89] and colorectal (*n* = 9; 10.2%) [7, 35, 39, 40, 43, 63, 73, 75, 91]. Thirteen studies (14.8%) included multiple primaries [6, 11, 26, 28, 32, 38, 45, 46, 54, 56, 57, 65, 67]. Most studies were retrospective (*n* = 81; 92.0%) and seven (8.0%) were prospective [15, 20, 47, 61, 65, 74, 78].

### Factors of variance

This paragraph summarizes published data on the different factors that determine the variance of response evaluations. We describe the different factors by tumor types. Percentage agreement and the Kappa coefficient are commonly used for comparing categorical responses. Bland–Altman analysis, correlation, ICC, and CV are commonly used for continuous data.

#### Manual measurement

We identified 19 studies that examined IOV related to manual measurements, all of which compared manual to semi-automatic methods. Many of these studies also investigated other influencing factors, such as 1D versus 3D measurements. When results could not be clearly separated (e.g., 1D manual vs 3D semi-automatic), we reported them only once to avoid duplication. With the exception of one study [39], all studies consistently found that manual measurements were associated with higher IOV than semi-automatic approaches.

*Lung carcinoma*: Awad et al [9] demonstrated that IOV between manual and semi-automatic decreased progressively from 1D (r2 = 0.63), to 2D (r2 = 0.87) to 3D (r2 = 0.96) diameters of 21 lung tumors. The IOV, however, remained excellent for both manual (intraclass correlation (ICC) > 0.98, coefficient of variation (CV) < 5.9%) and semi-automatic measurements (ICCs > 0.99, CV < 21.6%). Similar (intra- and interobserver) variability in semi-automatic measurements was reported by Bendtsen et al [10] on 10 patients using Bland–Altman (< 5.4%) and Concordance Correlation Coefficient (CCC > 0.99), and Dinkel et al [79] on 24 patients (ICC > 0.98), but their CV for manual measurements was two-to-three times higher. Moreover, they also found a bias in manual measurements, which were systematically smaller than semi-automatic ones.

*Other primary tumors:* Fabel et al [47] prospectively compared manual diameters and semi-automated volumes of 227 enlarged lymph nodes of 47 patients. Using Bland–Altman analysis, they found a limit of agreement (LoA) of 2.87 mm for manual 3D and 1.94 mm for semi-automatic 3D diameters. Similar trends were observed in relative differences, with 1.2% for semi-automatic 3D and 4.1% for manual 3D diameters. Further research from the same group reported faster evaluation times [16] and recommended using thin-slice reconstructions (≤ 3 mm) and medium soft tissue kernels for optimal results in automatic measurements [17].

Öztürk et al [52] found almost perfect intra- and interobserver agreement (ICC > 0.99) of both semi-automatic 3D and 1D measurements of 21 retroperitoneal lymph node metastases from testicular cancer. However, Folio et al [18] using Bland–Altman analysis, showed a decrease of IOV from 26.4% of 1D manual measurements to 8.9% of 3D semi-automatic measurements (mean percentage change) on *n* = 93 prostate cancer lung, liver, lymph nodes lesions.

In colorectal liver metastases, van Kessel et al [39] found semi-automated 3D measurements to have higher intra-reader variability (CV = 12.7%) compared to manual 1D (CV = 6.1%). A similar pattern was observed for interobserver agreement (14.9% 4.2%, 2.0%, respectively, using Bland–Altman relative measurement error). Semi-automated diameters did not significantly reduce variability in response classification either (kappa = 0.65 1D manual vs 0.55 automated) [39].

Bauknecht et al [67] found that semi-automated 3D measurements of 355 brain metastases on 75 MRI scans were more consistent than manual 1D measurements (27.8% to 33.0% LoA 21.4% to 23.3%). Li et al [26], in a group of 100 patients presented to five image analysts, showed that semi-automatic 3D measurements had higher agreement (ICC = 0.70 vs 0.63) but similar CV (0.33 vs 0.32) and Fleiss’ Kappa (0.59 vs 0.58) compared to 1D manual measurements. However, when presented with a second set of 100 patients, results improved in favor of semi-automatic measurements, suggesting the presence of a learning curve for automation software.

Monsky et al [64] found a good intra- and IOV (Wilcoxon *p* < 0.05 and ICC > 0.9, respectively) and reported no significant association between change in semi-automatic 3D and 1D measurements (*p* = 0.14) in 27 patients with liver tumors. On primary liver tumors, Bonekamp et al [71] showed semi-automatic 3D measurements reach a much lower interobserver variability (ICC = 0.85–1.00) than 1D manual measurements (ICC = 0.17). Kanaly et al [66] reported lower IOV for semi-automated 3D measurements (ICC = 0.97) compared to manual 1D measurements (ICC = 0.42) or 2D measurements (ICC = 0.61) in 13 glioblastoma patients. Dicken et al [15] showed that semi-automatic 3D measurements decreased IOV in measuring change over time of 30 renal cell carcinoma lung lesions, increasing the correlation between readers from 63.9% to 87.6%. However, it had a limited effect on their absolute values (from 95.6% to 99.4%). For 41 breast cancer patients, RECIST based on semi-automated differed from manual measurements in 27% of the cases, but did not reach significance [24]. See Table 1A for an overview of all studies.

**Table 1:** Overview of study characteristics stratified by comparison category

A. Manual versus (semi)-automated assessment									
Study	Year	Sample size	Study design	Imaging modality	Response criteria	Primary tumor	Treatment	Comparison	End point
Colombi et al	2015	47	Retrospective	CT	RECIST 1.1	NSCLC	Treatment for NSCLC	Manual axial longest diameter vs semi-automatic axial longest diameter vs volume of NSCLC lesions	Semi-automatic axial longest diameter and volume are more reproducible than manual axial longest diameter measurement (concordance correlation coefficient: 0.950 to 0.984) For 75% of the readers, a decrease of ≤ 70% in tumor volume was associated with a shorter survival (median survival: 11 months, *p* < 0.05; hazard ratio: 5–22.2, *p* < 0.05).
Awad et al	2012	15	Retrospective	CT	RECIST, WHO, 3D Volume	Lung carcinoma	Not mentioned	Segmentation algorithm to generate automated lung tumor measurement vs manual segmentation	Linear regression goodness-of-fit measures demonstrated significant correlations between manual and algorithmic results for 1D (r² = 0.63, *p* < 0.0001), 2D (r² = 0.87, *p* < 0.0001), and 3D measurements (r² = 0.96, *p* < 0.0001). The intra-observer intraclass correlation coefficients (ICC) indicated high reproducibility for both the algorithmic (0.989–0.995 for 1D, 0.996–0.997 for 2D, and 0.999–0.999 for 3D) and manual measurements (0.975–0.993 for 1D, 0.985–0.993 for 2D, and 0.980–0.992 for 3D).
Bendtsen et al	2011	10	Retrospective	CT	RECIST, Volume	Lung carcinoma	Treatment for lung carcinoma	Semi-automated approach for volumetric analysis of lung tumors vs line lengths	When comparing line length measurements to volumetric analysis, volumetric analysis offers greater sensitivity in evaluating treatment response. This also accounts for patients with advanced-stage cancer and complex lesions.
Dicken et al	2015	174	Prospective	CT	RECIST, Volume	Renal cell carcinoma	Sorafenib or IFN-2a	Manual study diameter (SDM) of the central readers using RECIST vs computer-aided volume measurements	The manual study diameters of two different readers showed a correlation of 95.6%, The effective diameter of computer-aided volume-equivalent sphere measurement showed a higher correlation: 99.4%. Evaluation of change rates showed a higher correlation for the effective diameter of the volume-equivalent sphere in comparison to the manual study diameter (87.6% vs 63.9%).
Dinkel et al	2013	24	Retrospective	CT, MRI	WHO, RECIST 1.1	Lung carcinoma	Prior to treatment	Manual vs semi-automated measurements of the tumor longest diameter	Semi-automated measurements demonstrated significantly higher intra-rater reliability compared to manual measurements for both longest diameter (intraclass correlation coefficients: 0.998 vs 0.986; *p* < 0.001) and area (0.995 vs 0.988; *p* = 0.032). Manual measurements of the tumor’s longest diameter were significantly smaller (*p* < 0.05) compared to semi-automated measurements.
Fabel et al	2011	50	Retrospective	CT	RECIST 1.1, Volume	Melanoma	Not mentioned	Manual measurement short-axis diameter of lymph node vs volumetric analysis using semi-automated software	Quality of segmentation by the software was assessed as acceptable (76–79%) by each reader. Semi-automated volumetric analysis allows fast (mean time spent: 38 s) segmentation of most lymph node metastases. Compared with RECIST, the interobserver-variability in baseline and follow-up is reduced (27.5% to 10.6%).
Fabel et al	2012	15	Retrospective	CT	RECIST 1.1, Volume	Melanoma	Not mentioned	Manual long axis diameters (LAD) as well as short-axis diameters (SAD) vs automated RECIST measurements.	Mean absolute percentage error (APE) for volumetric analysis ranged from 3.95% to 13.8%, increasing significantly with slice thickness. APE was not significantly different between LAD and SAD measurements. Automated RECIST showed a trend toward lower APE compared to manual measurements.
Folio et al	2013	50	Retrospective	CT	RECIST 1.1	Urogentical carcinoma	Treatment for metastatic bladder or prostate cancer	Manual vs lesion management application methods to measure the longest axis (or short axis for lymph nodes)	Measurement variability showed a mean percentage change of 8.9% with the lesion management application vs 26.4% for manual measurements.
Kanaly et al	2014	13	Retrospective	MRI	RECIST, MacDonald Criteria (2D), Volume	Glioblastoma	Surgery or biopsy or external beam radiation therapy or temozolomide chemotherapy	Semi-automated, volumetric method for quantifying enhancing tissue vs one-dimensional and two-dimensional measurements	Strong interobserver agreement was observed for RECIST (ICC = 0.42) and Macdonald (ICC = 0.61) criteria, but significantly higher with the automated method (ICC = 0.97). The volumetric approach showed the highest agreement for radiographic response (κ = 0.96), outperforming 2D (κ = 0.54) and 1D (κ = 0.46) methods.
Keil et al	2014	41	Retrospective	CT	RECIST 1.1	Mammacarcinoma	Systemic chemotherapy	Manual vs automated size assessment	Overall between-reader agreement was moderate (κ = 0.53) Readers selected the same target lesions in 25 cases and different ones in 16: same target lesions: 76% agreement on response (19/25) and different target lesions: 81% disagreement on response (13/16) (*p* < 0.001). Disagreement due to manual vs automated or 1D vs volumetric measurements was 27% and 15%, respectively.
Li et al	2022	200	Retrospective	CT	RECIST 1.1, WHO	Mixed	Anticancer treatment specific for primary tumor	Manual lesion diameter measurement vs computer-aided-contouring tool tumor measurement	The mean coefficient of variation (CV) for manual measurements remained stable (0.33 vs 0.32; *p* = 0.490), while CV for computer-aided contouring (CAC) tool measurements decreased significantly post-learning (0.24 → 0.19; *p* < 0.001).
Kessel et al	2012	22	Retrospective	CT	RECIST 1.0	Colorectal carcinoma	Chemotherapy	Manual diameter (MD) vs automatic diameter (AD) vs automatic volume (AV)	The intra-observer variability of measurements of the manual diameter (MD), automatic diameter (AD), and automatic volume (AV) was 6.05%, 4.28%, and 12.72%, respectively. The interobserver variability for MD, AD, and AV were 4.23%, 2.02%, and 14.86%, respectively. Agreement in response classification did not improve with automated methods: MD: κ = 0.653, AD: κ = 0.548, and AV: κ = 0.548.
Bauknecht et al	2010	75	Retrospective	MRI	Volume	Mixed	Not specified	Diameter vs semi-automated volume measurements of brain metastases	Interobserver variability of the RECIST diameter measurements: limits of agreement: ± 27.8% to ± 33.0% and unsigned mean difference: 0.2–2.5%. Interobserver variability of the volume measurements: limits of agreement: ± 21.4% to ± 23.3%, unsigned mean difference: 0.1–0.3%. Variability was significantly lower for volume vs diameter (*p* ≤ 0.001).
Fabel et al	2008	47	Prospective	CT	RECIST 1.1, Volume	Melanoma	Not mentioned	Manual length measurements in mm according to RECIST Vs Volume calculation in mL after manual segmentation Vs Volume calculation in mL after segmentation using the semi-automated software tool	Theemi-automated tool rated acceptable to excellent in 81% (reader 1) and 79% (reader 2). Median segmentation time was shorter for the semi-automated method compared to manual segmentation. Bland–Altman analysis showed significantly lower interobserver variability with semi-automated volumetry than with RECIST.
Monsky et al	2012	18	Prospective	MRI	RECIST	Sarcoma	Imatinib	Tumor volume and percent necrosis according to semi-automated segmentation vs histology and RECIST measurements	The median tumor necrosis percentage for histology was 71.9%, and for the semi-automated CE-MRI, it was 67.8%. In terms of accuracy, CE-MRI–based semi-automated necrosis measurements were statistically similar to histopathologic results.
Öztürk et al	2017	21	Retrospective	CT	RECIST 1.1, Volume	Urogenital carcinoma	Chemotherapy	Tumor response according to computerized volumetric analysis versus manual measurement according to RECIST criteria (version 1.1)	The intra- and interobserver variance was non-significant for both manual and volumetric measurements. In terms of reproducibility, volumetric analysis showed excellent agreement with ICC values > 0.99 for all observers. Minimal bias observed in both manual and volumetric modalities.
Bonekamp et al	2014	50	Retrospective	MRI	RECIST, EASL, Volume	Hepatocellular carcinoma	Intra-arterial therapy	Two semi-automated methods vs manual approach for functional, volumetric and morphologic parameters.	The interobserver agreement for apparent diffusion coefficient (ADC), arterial phase enhancement (AE) and portal venous phase enhancement (VE) for the semi-automated measurements was ICC = 0.830–0.974, and for the manual measurements, it was: ICC = 0.157–0.799. The interobserver agreement for tumor volume and axial size measurements for the semi-automated measurements was ICC = 0.854–0.996, and for the manual measurements, it was: ICC = 0.543–0.596.
Mattonen et al	2013	22	Retrospective	CT, MRI	RECIST 1.1. Volume	Lung carcinoma	Stereotactic ablative radiotherapy (SABR)	CT image feature analysis vs RECIST vs 3D Volume	The 3D volume and RECIST measures of consolidation could significantly distinguish recurrence from radiation induced lung injury from 15 months, with a mean 3D volume for recurrence of 30.1 ± 19.3 cm³ versus radiation induced lung injury (RILI) 5.1 ± 3.6 cm³ (*p* = 0.030). For RECIST size of recurrence and RILI, this was respectively 4.34 ± 1.13 cm and 2.63 ± 0.84 cm (*p* = 0.028).
Pirasteh et al	2021	82	Retrospective	CT, MRI	mRECIST for HCC LIRADS	Hepatocellular carcinoma	Drug-eluting beads transarterial chemoembolization (DEB-TACE)	Liver Imaging Reporting and Data System (LI-RADS) treatment response algorithm (LR-TR) vs mRECIST for HCC	The inter-reader agreement was moderate for LR-TR and mRECIST-overall: κ = 0.42–0.57 and substantial for mRECIST-target: κ = 0.62–0.66. There was no significant association between LR-TR or mRECIST and overall survival.

B. Selection target/measurable lesions									
Study	Year	Sample size	Study design	Imaging modality	Response criteria	Primary tumor	Treatment	Comparison	End point
Armato et al	2014	50	Retrospective	CT	mRECIST for mesothelioma	Mesothelioma	Not specified	Tumor thickness selection (visceral margin)	Systematic bias occurred where certain observers consistently measured thicker or thinner tumors compared to others. The overall interobserver variability across 170 lesions was 15.1% of the per-site mean (SD = 9.1%). For tumors with a size of 5–7.5 mm, the 95% CI for interobserver differences remained within RECIST thresholds.
Beaumont et al	2018	179	Retrospective	CT, MRI, PET	RECIST 1.1	Lung carcinoma	Cabazitaxel, topotecan	Average number of target and non-target lesions selected per patient for each local investigator (LI) and blinded independent central review (BICR)	The average number of target lesions per patient for the local investigators versus blinded independent central review was 2.9 vs 3.4 (*p* < 0.05), respectively.
Canals-Lambarri et al	2015	94	Retrospective	CT	WHO, RECIST 1.0	Mixed	Chemotherapy	Response of different solid types of lesions within each cancer type	Response patterns were similar between different tumor types. RECIST and WHO criteria overestimated volume changes, especially in intermediate response ranges. Including lymph nodes as target lesions led to distorted response evaluation.
Keil et al	2014	41	Retrospective	CT	RECIST 1.1	Mammacarcinoma	Chemotherapy	Target lesions selection	The overall between-reader agreement was moderate (κ = 0.53). When asked to select target lesions, there was agreement in 19/25 patients (76%) and disagreement in 13/16 (81%) (*p* < 0.001) if the same target lesions were selected. After dichotomizing responses (progressive vs non progressive), there was still disagreement due to manual vs automated measurements: 11/41 (27%) and unidimensional vs volumetric measurements: 6/41 (15%).
Krasovitsky et al	2022	47	Retrospective	CT	RECIST 1.1	Ovarian carcinoma	Treatment for advanced ovarian carcinoma	Target lesion selection	Lesion selection reproducibility was high: interobserver 0.91, intra-observer 0.93. Intra-observer reproducibility was perfect (1.0) for pelvic mass, hepatic, nodal, and other lesions. Lowest reproducibility was seen in peritoneal lesions (interobserver 0.76, intra-observer 0.69). Lesion measurement concordance was very good: interobserver ICC: 0.84, intra-observer ICC: 0.94. Interobserver differences accounted for 85% of total measurement variability.
Muenzel et al	2012	20	Retrospective	CT	RECIST 1.1	Mixed	Specific treatment for primary tumor	Target lesion measurement	Median intra-observer variability for single lesions: 4.9–9.6% (mean 5.9%). Median interobserver variability: 4.3–11.4% (mean 7.1%), across different time points, imaging systems, and observers.
Oubel et al	2015	11	Retrospective	CT	RECIST 1.1, VBR criteria	Lung carcinoma	Treatment for lung carcinoma	Effects of a consensual lesion selection on volume-based response (VBR) assessments	Volume-based response (VBR) with consensual target lesion selection: κ₍Fleiss₎ = 0.85 (SE = 0.091). With additional consensus on new lesions, κ₍Fleiss₎ increases to 0.95 (SE = 0.092). RECIST without consensus: κ₍Fleiss₎ = 0.72 (SE = 0.088). Differences were statistically significant (z-test).
Zhao et al	2014	30	Retrospective	CT	RECIST 1.0	Colorectal carcinoma	Treatment for metastatic colorectal carcinoma	Selection and measurement of target lesion	When three radiologists selected target lesions, total unique lesions were selected by all three in 33% of the cases, by two in 28%, and by one in 39% of the cases. Measurement variability with independent selection was unidimensional: 11%, bidimensional: 19%, and volumetric: 22%.
Moskowitz et al	2009	50	Retrospective	CT	RECIST 1.0	Not specified	Treatment for primary tumor	Impact of the number of lesions	Response assessments based on 5 vs 10 lesions showed minimal differences. Fewer than 5 lesions led to notable discrepancies compared to 10-lesion assessments. Using < 5 lesions tends to overestimate response and may misclassify patients. Measuring 5 lesions per patient appears sufficient for capturing treatment response.
Mercier et al	2020	189	Retrospective	MRI	RECIST 1.1	Colorectal carcinoma	Bevacizumab or vanucizumab on top of chemotherapy	Assess the heterogeneity across organs in response to treatment	Baseline tumor size varied significantly: liver lesions were over 2× larger than lung lesions. More durable response observed in lymph nodes and ‘other organs’ vs liver and lungs.

C. Dimensional comparison of measurable lesions									
Study	Year	Sample size	Study design	Imaging modality	Response criteria	Primary tumor	Treatment	Comparison	End point
Bendtsen et al	2011	10	Retrospective	CT	RECIST, Volume	Lung carcinoma	Treatment for lung carcinoma	Sensitivity of the volumetric analysis vs sensitivity of the RECIST line lengths on the same set of clinical images RECIST (1D) vs Volume	Clinical cases showed an intra-reader variability: ~ 5% (upper 95% CI: 14%) and inter-reader variability: ~ 5% (upper 95% CI: 19%). Volume measurements showed greater sensitivity than line lengths in assessing treatment response.
Colombi et al	2015	47	Retrospective	CT	RECIST 1.1	NSCLC	Treatment for NSCLC	Manual axial longest diameter vs semi-automatic axial longest diameter vs volume of NSCLC lesions RECIST 1.1. (1D) manual VS RECIST 1.1. (1D) manual (semi-automated) VS Volume	The semi-automatic measurements were more reproducible than the manual measurements: axial longest diameter: CCC 0.980–0.987; VC% 6–7.3%, volume: CCC 0.974–0.991; VC% 5.6–9.5%, manual diameter: CCC 0.950–0.984; VC% 6.4–11.7%. The RECIST categories did not stratify survival outcomes.
Dicken et al	2015	174	Prospective	CT	RECIST, Volume	Renal cell carcinoma	Sorafenib or IFN-2a	Manual study diameter (SDM) of the central readers using RECIST vs computer-aided (EDM) volume measurements Manual diameter (1D) VS Volume	The reader correlation for SDM (standard diameter measurements) and EDM (enhanced diameter measurements) was 95.6% and 99.4%, respectively. Correlation with complete response (CR) was SDM-CR: 63.9% and EDM-CR: 87.6%.
Fabel et al	2011	50	Retrospective	CT	RECIST 1.1, Volume	Melanoma	Not mentioned	Manual measurement short-axis diameter of lymph node vs volumetric analysis using semi-automated software Manual diameter (1D) vs Volume	Software performance: 76–79% of segmentations rated acceptable to excellent by readers. The variability in changes of the effective diameter was 10.6%, and for the RECIST maximum diameter: 27.5% The semi-automated volumetric analysis enabled rapid segmentation of most lymph node metastases and reduced interobserver variability, especially in baseline and follow-up measurements compared to RECIST.
Fabel et al	2012	15	CT	Retrospective	RECIST 1.1, Volume	Melanoma	N/A	To investigate the reproducibility of semi-automated volumetric analysis of lymph node metastases as a function of both slice thickness and reconstruction kernel. In addition, manual long axis diameters (LAD) as well as short-axis diameters (SAD) were compared to automated RECIST measurements. RECIST 1.1. VS Volume	The mean absolute percentage error (APE) for volumetric analysis was 3.95–13.8%. This increased with slice thickness. Reconstruction kernel differences were not significant, but the trend favored the middle soft tissue kernel. There was no significant difference between automated and manual LAD/SAD (RECIST 1.0/1.1). Poor segmentation was mainly seen with thicker slices (3–5 mm).
Hajkova et al	2022	61	Prospective	CT	RECIST mRECIST for HCC Volumetric assessment	Hepatocellular carcinoma	Transarterial chemoembolization (TACE)	Prognostic value of RECIST vs mRECIST for HCC and Volumetric analysis RECIST vs mRECIST for HCC VS Volume	The volumetric assessment was a significant prognostic factor for overall survival (*p* < 0.01) and progression-free survival (*p* < 0.001). RECIST and mRECIST were not significant prognostic factors.
Kanaly et al	2014	13	MRI	Retrospective	RECIST, MacDonald Criteria (2D), Volume	Glioblastoma	Surgery or biopsy or external beam radiation therapy or temozolomide chemotherapy	Semi-automated, volumetric method for quantifying enhancing tissue vs one-dimensional and two-dimensional measurements 1D vs 2D vs Volume	The inter-reader agreement (ICC) for RECIST (1D), Macdonald (2D), and automated volumetric was 0.42, 0.61, and 0.97, respectively. The radiographic response agreement (kappa) was: volumetric: 0.96, 2D: 0.54, and 1D: 0.46
Keil et al	2014	41	Retrospective	CT	RECIST 1.1	Mammacarcinoma	Chemotherapy	Computer-assisted diagnosis system for automated unidimensional vs 3-dimensional assessment of target lesions. 1D vs 3D	The overall between-reader agreement was moderate (κ = 0.53). Selection of different target lesions was associated with an 81% rate of disagreement (13/16) (*p* < 0.001). Disagreement rates due to manual versus automated or unidimensional versus volumetric size measurements were less important (11/41 and 6/41; 27% and 15%, respectively).
Lubner et al	2017	105	Retrospective	CT	RECIST 1.1	Metastatic colorectal cancer	Chemotherapy	Volumetric Versus Unidimensional Measures of Metastatic Colorectal Cancer in Assessing Disease Response 1D VS Volume	Overall survival prediction: 1D and 3D assessments showed comparable performance (Cox and Kaplan–Meier analyses). Response category discrimination: Both methods effectively distinguished PD vs SD/PR. But neither clearly differentiated SD vs PR. 1D and 3D showed similar intra- and interobserver variability using semi-automated methods (Bland–Altman).
Rothe et al	2013	45	Retrospective	CT	RECIST 1.1. Volume	Mixed	Treatment for tumor type	RECIST 1.1 vs 3D Volume	RECIST-based volumes were significantly higher than other methods (*p* < 0.001), with relative differences of 0.4–41.1% (ANOVA). Intra-observer variability was significantly higher for RECIST and threshold-based segmentation: 3.6–32.8%. In follow-up, 3D algorithms vs RECIST 1.1 led to discordant response classifications in 10–21% of patients.
Schoot et al	2013	64	Retrospective	CT, MRI	RECIST 1.1. EPsGG	Sarcoma	Chemotherapy	1D vs 3D	The interobserver agreement for EpSSG and RECIST was κ = 0.565 and κ = 0.592 (both moderate). Interobserver variation affected potential treatment decisions in 9 patients (14%) for EpSSG and 11 patients (17%) for RECIST EpSSG vs RECIST: 13 discrepant classifications (20%). This affected treatment decisions in 5 patients (8%).
Sohns et al	2010	68	Retrospective	CT	RECIST WHO Volume (region growing criteria)	Lung carcinoma	Treatment for SCLC/NSCLC	Unidimensional (1D) vs bidimensional (2D) vs volume	RECIST and WHO criteria showed high agreement (κ = 0.76–0.82). There were discrepancies for lesions with moderate size increases, where WHO more frequently classified lesions as progressive. Agreement was highest between RECIST and region growing criteria (RGC) (κ = 0.79–0.87). Reproducibility was highest with RGC (96%), followed by RECIST and WHO (both 95%). Relative measurement error was lowest for RECIST (2.82%).
Van Kessel et al	2012	22	Retrospective	CT	Manual diameter, automatic diameter, automatic volume	Colorectal carcinoma	Chemotherapy	Automatic diameter vs volume measurements 1D vs Volume	The intra-observer variability for the measurement of the manual diameter (MD), automatic diameter (AD), and automatic volume (AV) was 6.05%, 4.28%, and 12.72%, respectively. The interobserver variability for the measurement of MD, AD, and AV was 4.23%, 2.02%, and 14.86%, respectively. The agreement in the response classification was: MD: κ = 0.653, AD: κ = 0.548, and AV: κ = 0.548.
Steger et al	2011	13	Retrospective	CT	RECIST 1.1., Volume	Oral carcinoma	N/A	Long and the short axis in the axial plane VS three-dimensional (3D) assessment 1D vs Volume	The interobserver variability was 10% for all measured lengths and 16% for computed volume. The RECIST 1.1 short-axis measurement is more robust due to less sensitivity to spatial orientation. But combining both axes yields a better volume estimate than using the short axis alone.
Wulff et al	2013	56	Retrospective	CT	RECIST 1.1., Volume	Not specified	Treatment specific for tumor type	RECIST 1.1 vs volumetric assessment 1D vs Volume	The interobserver variability for manual measurements was: 6.3% (IQR 4.6%) and for volumetric measurements: 4.1% (IQR 4.4%) (*p* < 0.05). The Fleiss’ kappa for response classification for manual and volumetric measurements was 0.7558 and 0.7623, respectively.
Zimmerman et al	2021	50	Retrospective	CT	RECIST 1.1., Volume	Mixed	Treatment specific for tumor type	Unidimensional vs Volume	The same response classification across 3 readers was measured when using uRECIST in 33/50 patients (66%). When using vRECIST, this was 42/50 patients (84%) and 44/50 patients (88%). The inter-reader agreement improved from 0% (uRECIST) to 36% (vRECIST).
Jiang et al	2017	67	Retrospective	CT	RECIST 1.1., WHO	Lung carcinoma	Treatment for lung carcinoma	1D vs 2D vs Volume	Volumetric technique showed the lowest measurement variability vs unidimensional and bidimensional methods. Tumor characteristics (object effect) were the main source of variability; rater effect was minimal. Segmentation and tumor size influenced variability. A nonlinear mathematical relationship was found between tumor size and volumetric variability.
King et al	2020	57	Retrospective	CT, MRI	mRECIST for HCC, LIRADS TR, pEASL	HCC	Yttrium-90 radioembolization	1D vs 3D	The inter-reader agreement was: mRECIST: Fair (K = 0.43, 0.34 at first and second follow-up) and LIRADS TR: Moderate (K = 0.48, 0.53). The inter-criterion agreement was: mRECIST vs qEASL: Moderate to substantial (r = 0.41–0.65, 0.54–0.60). LIRADS TR vs qEASL: Good correlation across all readers (K = 0.45–0.78, 0.39–0.77).
Villemaire et al	2011	7	Retrospective	CT	RECIST 1.1.	Renal cell carcinoma	Treatment with anti-neoplastic therapy	Accuracy and reproducibility 1D, 2D, 3D	All methods showed high accuracy for the spherical phantoms. For the irregular phantoms, only 3D measurements aligned well with ground truth. The intra- and interobserver reproducibility was high for all methods (ICC > 0.900).
Zhao et al	2013	30	Retrospective	CT	RECIST 1.0, Volume	Mixed	Treatment specific for tumor type	Intra- and inter-reader variability 1D, 2D, 3D using manual and computer-aided method	The maximum intra-/inter-reader variability for the manual measurements was: 1D: 6.9%/9.0% and 2D: 12.3%/18.0%. For the computer-assisted method (CAM) this was 1D: 5.4%/9.3%, 2D: 11.3%/18.8% and volume (3D): 9.3%/18.0% The 95% reference ranges for intra-reader % difference was: manual 1D: (−15.5%, 25.8%) and manual 2D: (−27.1%, 51.6%).
Huang et al	2019	93	Retrospective	CT, MRI	RECIST 1.1., RANO, Volume	Meningeoma	Bevacizumab and trastuzumab	1D vs 2D vs Volume	The tumor progression thresholds and survival for volumetric increase ≥ 40% and 1D increase ≥ 10 mm at 6–12 months showed the strongest association with OS: HR = 2.58 and 3.24, respectively (*p* < 0.01). The volume thresholds > 40% did not correlate with survival. Tumor shrinkage was not associated with OS (*p* > 0.09). The interobserver agreement (Kappa) was 1D: 0.49, 2D: 0.46, and volume: 0.52.
Nishino et al	2013	57	Prospective	CT	RECIST 1.1. iRECIST	Melanoma	Salvage Radioembolization Using 90Y-Loaded Resin Microsphere	1D vs 2D	Percent change concordance between unidimensional and bidimensional irRC: Spearman r = 0.953–0.965 (across first to fourth follow-up) Best immune-related response: high agreement between methods (weighted κ = 0.881) Time to progression (TTP) was similar between methods: bidimensional: 70% progression-free at 6 months, and unidimensional: 81% progression-free at 6 months
Zhao et al	2014	29	Retrospective	CT	RECIST 1.0	Colorectal carcinoma	N/A	Variability in relative change of tumor measurements between 1D, 2D and volume	Variability in relative change (with independent selection): unidimensional: 11%, bidimensional: 19%, and volumetric: 22%. Variability when measuring the same lesions: unidimensional: 8%, bidimensional: 14%, and volumetric: 12%.
Zhao et al	2022	141	Prospective	CT	RECIST	Mixed (pulmonary lesions)	Not specified	Deep learning image reconstruction-based ultra-low-dose CT vs contrast-enhanced CT to measure diameter of pulmonary lesions and lymph nodes	Measurement variation between deep learning image reconstruction-based ultra-low-dose CT versus contrast-enhanced CT for pulmonary lesions was 2.2% (95% CI: 1.7–2.6%), and for lymph nodes it was 1.4% (95% CI: 1.0–1.9%).

D. Inter- and intra-observer variability in repeated lesion measurements using the same imaging modality									
Study	Year	Sample size	Study design	Imaging modality	Response criteria	Primary tumor	Treatment	Comparison	End point
Oxnard et al	2011	30	Retrospective	CT	RECIST 1.1	Lung carcinoma	Treatment for lung cancer	Repeat CT scans performed within 15 min of each other	84% of measurement changes were within ± 10% 3% met RECIST progression criteria (≥ 20% increase). Smaller lesions showed greater variability in percent change (*p* = 0.005).

E. Different tumor response evaluation criteria									
Study	Year	Sample size	Study design	Imaging modality	Response criteria	Primary tumor	Treatment	Comparison	End point
Hajkova et al	2022	61	Prospective	CT	RECIST 1.1 mRECIST for HCC Volumetric assessment	Hepatocellular carcinoma	Transarterial chemoembolization (TACE)	Prognostic value of RECIST 1.1 vs mRECIST for HCC and Volumetric analysis	Volumetric assessment was a prognostic factor for overall survival (*p* < 0.01) and progression-free survival (*p* < 0.001). In contrast with RECIST and mRECIST.
Khoker et al	2015	151	Retrospective	Callipers and tape measure	WHO RECIST RECIST-Breast	Mammacarcinoma	Primary chemotherapy/Neoadjuvant chemotherapy (NACT)	WHO vs RECIST vs RECIST-Breast	RECIST showed 94% concordance and RECIST-B, with a lower progressive disease threshold (≥ 10% vs ≥ 20%), showed 97% concordance with WHO criteria
Rothe et al	2013	45	Retrospective	CT	RECIST 1.1 3D Volume	Mixed (liver metastasis from pancreatic and colon cancer)	Systemic anticancer treatment	3D vs RECIST for size measurement and response assessment	RECIST-based volumes were significantly higher than other methods (ANOVA, *p* < 0.001), with differences of 0.4–41.1%. Intra-observer variability was higher for RECIST and threshold-based segmentation: 3.6–32.8% and lower for slice segmentation: 0.4–13.7%. It was the lowest for seeded region growing: 0.6–10.8% (*p* < 0.001). Response classification discordance between 3D algorithms and RECIST 1.1 was observed in 10–21% of patients during follow-up.
Sato et al	2013	21	Retrospective	CT	RECIST 1.1 mRECIST for HCC	Hepatocellular carcinoma	Transcatheter arterial chemoembolization	RECIST 1.1 vs mRECIST for HCC	The response rates for mRECIST were: CR = 56.9%, overall response = 79.7% and for RECIST 1.1: CR = 9.2%, overall response = 43.1%. Reproducibility for mRECIST showed almost perfect agreement, and for RECIST 1.1, it showed substantial agreement.
Schoot et al	2013	64	Retrospective	CT, MRI	EpSSG RECIST 1.1	Sarcoma	Chemotherapy	EpSSG vs RECIST 1.1	The interobserver agreement for EpSSG and RECIST was κ = 0.565 and κ = 0.592, respectively. The EpSSG vs RECIST showed 13 discrepant classifications (20%), evenly split between under- and overestimation.
Seyal et al	2015	103	Retrospective	CT	RECIST 1.1 mRECIST for HCC	Hepatocellular carcinoma	Transarterial Radioembolization Therapy	RECIST 1.1 vs mRECIST for HCC	The mRECIST Intra-observer agreement was good (κ = 0.70), and the interobserver agreement was moderate (κ = 0.56). mRECIST detected significantly more early responses: early follow-up response rate for mRECIST was 40.8% and for RECIST: 3.9% (*p* = 0.025).
Suzuki et al	2010	39	Retrospective	CT	RECIST 1.1 WHO	Mixed (breast cancer, colorectal cancer)	Various anticancer treatments	RECIST 1.1 vs WHO	Interobserver agreement was: RECIST: κ = 0.53 (95% CI: 0.33–0.72) and WHO criteria: κ = 0.60 (95% CI: 0.39–0.80) Intra-observer agreement for RECIST and WHO is κ = 0.76–0.96 and κ = 0.86–0.91, respectively.
Tovoli et al	2018	115	Retrospective	CT, MRI	RECIST 1.1 mRECIST for HCC RECICL	Hepatocellular carcinoma	Sorafenib	RECIST 1.1 vs mRECIST for HCC vs RECICL	Response concordance among expert operators was RECIST 1.1: κ = 0.840, mRECIST: κ = 0.871, and RECICL: κ = 0.819.
Wulff et al	2013	56	Retrospective	CT	RECIST 1.1 Volume measurement	Mixed	Various anticancer treatments	RECIST 1.1 vs Volume measurement	Interobserver variability for manual measurements was 6.3% (IQR 4.6%) and for volumetric measurements: 4.1% (IQR 4.4%) (*p* < 0.05). Categorical agreement (Fleiss’ kappa) for manual and volumetric measurements was κ = 0.7558 and κ = 0.7623, respectively. There was no significant difference between methods in response classification agreement.
Zheng et al	2021	30	Retrospective	CT	iRECIST RECIST 1.1	Renal cell carcinoma	PD-1 inhibitor (immunotherapy)	iRECIST vs RECIST 1.1	The objective response rate for iRECIST was 50% (95% CI: 32.1–67.9), and for RECIST 1.1 it was 30% (95% CI: 13.6–46.4), *p* < 0.001.
Kim et al	2015	113	Retrospective	CT	mRECIST EASL	Hepatocellular carcinoma	Chemo-embolization	mRECIST for HCC vs EASL	Baseline measurements (between two observers): CCC: 0.992 (bidimensional), 0.988 (unidimensional), and mean differences: −0.455 cm (bidimensional), 0.079 cm (unidimensional) Follow-up measurement changes: CCC: 0.865 (bidimensional), 0.877 (unidimensional), and mean % difference: −9.715% (bidimensional), −9.320% (unidimensional) Tumor count agreement: kappa = 0.942 Treatment response agreement: kappa = 0.941 (all criteria) There was perfect agreement (κ = 1.000) when assessing ≤ 2 target lesions.
King et al	2020	57	Retrospective	CT, MRI	mRECIST for HCC LIRADS TR pEASL	Hepatocellular carcinoma	Transcatheter arterial yttrium-90 Radioembolization (TARE)	mRECIST for HCC vs LIRADS TR vs pEASL	The inter-reader agreement for mRECIST was fair (K = 0.43, 0.34 at first and second follow-up) and for LIRADS TR it was moderate (K = 0.48, 0.53) The inter-criterion agreement for mRECIST vs qEASL was moderate to substantial (r = 0.41–0.65 and 0.54–0.60), and for LIRADS TR vs qEASL it had a good correlation (K = 0.45–0.78 and 0.39–0.77) LIRADS TR was the most accurate for the prediction of histopathological necrosis.
Plathow et al	2008	50	Retrospective	CT, MRI	RECIST mRECIST pleural mesothelioma Volume	Mesothelioma	Chemotherapy	RECIST 1.0 vs mRECIST for mesothelioma vs Volume	Interobserver variability for modified RECIST was κ = 0.9–1.0 and for RECIST: κ = 0.6–1.0.
Ghosn et al	2021	37	Retrospective	CT, MRI	RECIST 1.1 mRECIST for HCC EASL vRECIST qEASL	Hepatocellular carcinoma	Transcatheter arterial yttrium-90 radioembolization (TARE)	RECIST 1.1 vs mRECIST for HCC vs EASL vs qEASL	Multivariate analysis showed a significant association with overall survival: RECIST 1.1: HR = 0.26 (95% CI: 0.09–0.75, *p* = 0.01), mRECIST: HR = 0.22 (95% CI: 0.08–0.59, *p* = 0.003), EASL: HR = 0.22 (95% CI: 0.07–0.63, *p* = 0.005), and qEASL: HR = 0.30 (95% CI: 0.12–0.80, *p* = 0.02). The interobserver reproducibility was the highest for RECIST 1.1.
Hakazoki	2019	26	Retrospective	CT	RECIST 1.1 ITMIG	Thymic carcinoma	Chemotherapy	RECIST 1.1 vs ITMIG	All 26 patients had the same best overall response using ITMIG criteria. There was no significant difference (*p* = 0.993) between the median time to progression for RECIST vs ITMIG-modified criteria.
Pirasteh et al	2021	80	Retrospective	CT, MRI	LI-RADS mRECIST for HCC	Hepatocellular carcinoma	Drug-eluting beads transarterial chemoembolization (DEB-TACE)	LI-RADS vs mRECIST for HCC	The inter-reader agreement for LIRADS and mRECIST was moderate κ = 0.42–0.57. It was higher for mRECIST κ = 0.62–0.66. There was no association with overall survival for LIRADS or mRECIST.
Schvartsman et al	2017	28	Retrospective	CT, MRI	RECIST 1.1, Choi	Gastrointestinal stromal tumor	Regorafenib	RECIST 1.1 vs Choi	Partial response rate for regorafenib was 29% for the Choi criteria and 4% for the RECIST criteria.
Shady et al	2016	26	Retrospective	PET-CT	RECIST 1.1, Tumor attenuation criteria, Choi criteria, EORTC PET criteria	Colorectal carcinoma	Salvage radioembolization using 90Y-loaded resin microspheres	RECIST 1.1 vs tumor attenuation criteria vs Choi criteria vs EORTC PET criteria	There was a partial response among 25 patients: EORTC PET criteria: 14, Choi criteria: 15, tumor attenuation criteria: 13 and RECIST 1.1: 2 EORTC PET, Choi, and attenuation criteria: were significant predictors for PFS.
Nishino et al	2014	90	Retrospective	CT	RECIST 1.1, iRECIST	Melanoma	Immunotherapy (ipilimumab)	RECIST 1.1 vs iRECIST	Median time-to-progression was 26.9 months (95% CI: 9.1–NA), this was consistent across both assessments. Interobserver agreement was high, with concordance correlation coefficients > 0.98.

F. Different imaging techniques									
Study	Year	Sample size	Study design	Imaging modality	Response criteria	Primary tumor	Treatment	Comparison	End point
Hajkova et al	2022	61	Prospective	CT	RECIST mRECIST for HCC Volumetric assessment	HCC	Treatment for HCC	Prognostic value of RECIST vs mRECIST for HCC and Volumetric analysis	Volumetric assessment was a significant prognostic factor for: overall survival (*p* < 0.01) and progression-free survival (*p* < 0.001). RECIST and mRECIST measurements were not a significant prognostic factor.
Khoker et al	2015	151	Retrospective	Callipers and tape measure	WHO RECIST RECIST-Breast	Mammacarcinoma	Primary chemotherapy/Neoadjuvant chemotherapy (NACT)	WHO vs RECIST vs RECIST-Breast	RECIST showed 94% concordance, reclassifying 9 of 151, leading to underestimation and a delayed PD diagnosis. RECIST-Breast showed 97% concordance with WHO criteria, reclassifying only 4 patients.
Rothe et al	2013	45	Retrospective	CT	RECIST 1.1 3D Volume	Mixed (liver metastasis from pancreatic and colon carcinoma)	Systemic anticancer treatment	3D vs RECIST 1.1 for size measurement and response assessment	ANOVA showed that volumes calculated using RECIST were significantly higher than those from other methods (*p* < 0.001). Intra-observer variability was significantly higher for RECIST and threshold-based segmentation (3.6–32.8%) compared to slice segmentation (0.4–13.7%) (*p* < 0.001).
Sato et al	2013	21	Retrospective	CT	RECIST 1.1 mRECIST for HCC	Hepatocellular carcinoma	Transcatheter arterial chemoembolization	RECIST 1.1 vs mRECIST for HCC	In the inter-criteria reproducibility study, mRECIST showed higher complete response (CR) (56.9%) and response rates (79.7%) compared to RECIST 1.1 (CR: 9.2%, response: 43.1%). In inter- and intra-observer reproducibility, mRECIST demonstrated ‘almost perfect agreement,’ while RECIST 1.1 showed ‘substantial agreement.’
Schoot et al	2013	64	Retrospective	CT, MRI	EpSSG RECIST 1.1	Sarcoma	Chemotherapy	EpSSG vs RECIST 1.1	Interobserver agreement was moderate for both EpSSG (κ = 0.565) and RECIST (κ = 0.592) Comparison of EpSSG and RECIST showed 13 discrepant response classifications (20%), evenly split between under- and overestimation. This impacted treatment decisions in 5 patients (8%).
Seyal et al	2015	103	Retrospective	CT	RECIST 1.1 mRECIST for HCC	HCC	Transarterial radioembolization therapy	RECIST 1.1 vs mRECIST for HCC	mRECIST showed good intra-observer (κ = 0.70) and moderate interobserver (κ = 0.56) agreement. In early follow-up, mRECIST showed a significantly higher response rate than RECIST (40.8% vs 3.9%; *p* = 0.025).
Suzuki et al	2010	39	Retrospective	CT	RECIST 1.1 WHO	Mixed (breast cancer, colorectal cancer)	Various anticancer treatments	RECIST 1.1 vs WHO	The interobserver agreement was moderate for RECIST (κ = 0.53, 95% CI: 0.33–0.72) and WHO criteria (κ = 0.60, 95% CI: 0.39–0.80). Intra-observer agreement ranged from 0.76–0.96 (RECIST) and 0.86–0.91 (WHO).
Tovoli et al	2018	115	Retrospective	CT, MRI	RECIST 1.1 mRECIST for HCC RECICL	Hepatocellular carcinoma	Sorafenib	RECIST 1.1 vs mRECIST for HCC vs RECICL	Overall response concordance between expert operators was good across all criteria: • RECIST 1.1: κ = 0.840 • mRECIST: κ = 0.871 • RECICL: κ = 0.819
Wulff et al	2013	56	Retrospective	CT	RECIST 1.1 Volume measurement	Mixed	Various anticancer treatments	RECIST 1.1 vs Volume measurement	Interobserver variability was lower for volumetric measurements (4.1%, IQR 4.4%) than manual (6.3%, IQR 4.6%) (*p* < 0.05, corrected). Mean interobserver differences in diameter change at first follow-up were larger (−9.715% for bidimensional, −9.320% for unidimensional). Kappa values between observers: • Tumor count: κ = 0.942 • Treatment response: κ = 0.941 (both criteria) • Two target lesions: κ = 1.000 (both criteria) Fleiss’ kappa showed no significant difference in response classification between manual (0.7558) and volumetric (0.7623) methods.
Zheng et al	2021	30	Retrospective	CT	iRECIST RECIST 1.1	Renal cell carcinoma	PD-1 inhibitor (immunotherapy)	iRECIST vs RECIST 1.1	Objective response rate was higher with iRECIST (50%, 95% CI: 32.1–67.9) than with RECIST 1.1 (30%, 95% CI: 13.6–46.4). Median time-to-progression was longer with iRECIST (not reached) vs RECIST 1.1 (170 days; *p* = 0.04). Tumor response assessments differed significantly between the two criteria (*p* < 0.001).
Kim et al	2015	113	Retrospective	CT	mRECIST EASL	HCC	Chemo-embolization	mRECIST for HCC vs EASL	Concordance correlation coefficients (CCC) at baseline between two observers were: • Bidimensional diameters: 0.992 • Unidimensional diameters: 0.988 CCC for changes at follow-up were lower: • Bidimensional: 0.865 •Unidimensional: 0.877 Conclusion: There was high agreement at baseline, with reduced concordance for follow-up changes.
King et al	2020	57	Retrospective	CT, MRI	mRECIST for HCC LIRADS TR pEASL	HCC	Transcatheter arterial yttrium-90 Radioembolization (TARE)	mRECIST for HCC vs LIRADS TR vs pEASL	Inter-reader agreement for mRECIST was fair (κ = 0.43 and 0.34 at first and second follow-up, respectively). For LI-RADS TR, agreement was moderate (κ = 0.48 and 0.53). Inter-criterion agreement between mRECIST and qEASL ranged from moderate to substantial (correlation 0.41–0.65 at first follow-up, 0.54–0.60 at second). LI-RADS TR showed good agreement with qEASL across readers (κ = 0.45–0.78 at first, 0.39–0.77 at second follow-up). qEASL was most effective in predicting tumor-free survival
Plathow et al (ALSO CT vs MRI)	2008	50	Retrospective	CT, MRI	RECIST mRECIST pleural mesothelioma Volume	Mesothelioma	Chemotherapy	RECIST vs mRECIST pleural mesothelioma vs Volume	Modified RECIST showed higher accuracy than RECIST in classifying patient responses Interobserver agreement was superior for modified RECIST.
Ghosn et al	2021	37	Retrospective	CT	RECIST 1.1 mRECIST for HCC EASL vRECIST qEASL	HCC	Transcatheter arterial yttrium-90 radioembolization (TARE)	RECIST 1.1 vs mRECIST for HCC vs EASL vs vRECIST vs qEASL	Multivariate analysis showed that RECIST 1.1, mRECIST, EASL, and qEASL all significantly differentiated overall survival between responders and nonresponders (HR range: 0.22–0.30; *p* < 0.05). RECIST 1.1 demonstrated the highest interobserver reproducibility.
Hakazoki	2019	26	Retrospective	CT	RECIST 1.1 ITMIG	Thymic carcinoma	Chemotherapy	RECIST 1.1 vs ITMIG	All patients had identical best overall responses using ITMIG criteria. Median time to progression: • RECIST: 5.5 months (95% CI: 3.8–8.6) • ITMIG-modified: 7.0 months (95% CI: 3.8–9.3) • No significant difference (*p* = 0.993).
Pirasteh et al	2021	80	CT, MRI	Retrospective	LI-RADS mRECIST for HCC	HCC	Drug-eluting beads transarterial chemoembolization (DEB-TACE)	LI-RADS vs mRECIST for HCC	Inter-reader agreement was moderate for LR-TR and mRECIST-overall (κ = 0.42–0.57), substantial for mRECIST-target (κ = 0.62–0.66), and consistent across all, experienced, and less-experienced readers. LI-RADS and mRECIST-target responses were not significantly associated with overall survival (*p* > 0.05). mRECIST-overall response was significantly associated with overall survival when assessed by all readers (*p* = 0.02) and experienced readers (*p* = 0.03).
Schvartsman et al	2017	28	CT, MRI	Retrospective	RECIST 1.1 Choi	Gastrointestinal stromal tumor	Regorafenib	RECIST 1.1 vs Choi	Partial response rate by Choi criteria (29%) was notably higher than by RECIST (4%), similar to findings with imatinib.
Shady et al	2016	26	PET-CT	Retrospective	RECIST 1.1 tumor attenuation criteria Choi criteria EORTC PET criteria	Colorectal carcinoma	Salvage radioembolization using 90Y-loaded resin microspheres	RECIST 1.1 vs tumor attenuation criteria vs Choi criteria vs EORTC PET criteria	Intraclass correlation coefficients (ICC) were high for baseline scans (0.95) and response evaluation (0.980). Liver PFS was significantly predicted by responses per EORTC PET (*p* < 0.001), Choi criteria (*p* < 0.001) and tumor attenuation (*p* = 0.01).

G. Different imaging techniques									
Study	Year	Sample size	Study design	Imaging modality	Response criteria	Primary tumor	Treatment	Comparison	End point
Biederer et al	2009	4 porcine lungs	Prospective	4D-CT, 4D-MRI, 4D-CBCT	RECIST 1.0	Not specified	No treatment	4D-CT vs 4D-MRI vs 4D-CBCT	Lesions appeared significantly larger on MRI: 2.06/1.95 cm (*p* < 0.05 vs CT), MRI vs CT/CBCT: 1.47/1.28 cm (*p* < 0.05), and CBCT: 1.86/1.83 cm (*p* < 0.05 vs CT). Interobserver variability coefficients (VC) for lesion size were CT: 2.54–4.47%, 4D-CT: 2.29–4.48%, MRI: 5.44–6.22%, and CBCT: 4.86–6.97%.
Huh et al	2018	44	Retrospective	CT	RECIST 1.1	Neuro-endocrine tumor	Chemotherapy, surgery, radiofrequency ablation, or transarterial chemoembolization	Pre-contrast, arterial phase, and portal venous phase	Interobserver agreement (limits of agreement, LOA) was best on pre-contrast CT: −6.1 to 5.7 mm, portal venous phase (PVP): −7.9 to 7.1 mm, and arterial phase (AP): −8.5 to 7.4 mm.
Moalla et al	2017	54	Retrospective	CT, MRI	RECIST 1.1	Neuro-endocrine tumor	Treatment-naïve or under somatostatin analogues	Triphasic abdominal-CT vs liver MRI	Intra-observer variability was lower than interobserver variability. T2-weighted (T2W) MRI and portal-phase CT showed the lowest measurement variability. T2W MRI had the best intra- and interobserver reproducibility. MRI was more reproducible than CT.
Oxnard et al	2011	30	Retrospective	CT	RECIST 1.1	Lung carcinoma	Treatment for lung cancer	Repeat CT scans performed within 15 min of each other	84% of measurement changes were within ± 10% 3% met RECIST progression criteria (≥ 20% increase). Smaller lesions showed greater variability in percent change (*p* = 0.005).
Luersen et al	2016	30	Retrospective	MRI	RECIST	Neuro-endocrine tumor	Treatment for neuro-endocrine tumor	Various MR series: T1 (in/out-of-phase, T2, DWI, Pre- and Post-Gd-EOB-DTPA 3D gradient echo) (4 phases plus 20 min (HBP-Gd))	Hepatobiliary phase with gadoxetate (HBP-Gd) showed the lowest interreader variability. No significant differences in interreader variability were observed across imaging series. Sensitivity of lesion detection varied significantly across some sequences when compared to HBP-Gd.
Plathow et al	2008	50	Retrospective	CT, MRI	mRECIST pleural mesothelioma, RECIST	Mesothelioma	Chemotherapy	MRI vs CT	Modified RECIST showed superior interobserver agreement compared to RECIST (κ = 0.9–1.0 vs 0.6–1.0). Modified RECIST offers a highly accurate and reproducible method for evaluating early therapy response in MPM.
Ma et al	2020	114	Retrospective	CT, MRI	RECIST 1.1	Pancreatic adenocarcinoma	Not specified	CT versus MRI versus measurements of resected pathologic specimens	Mean tumor size differences compared to pathology were: CT: 4.3 mm (ICC = 0.67) and MRI: 5.8 mm (ICC = 0.65). Tumors > 30 mm were underestimated on CT and MRI compared to pathology. Discrepancies between imaging and pathological tumor sizes varied significantly by T stage (*p* < 0.001).

H. Radiologist versus other reviewer									
Study	Year	Sample size	Study design	Imaging modality	Response criteria	Primary tumor	Treatment	Comparison	End point
Gouel et al	2023	90	Retrospective	CT	RECIST 1.1	Mammacarcinoma	Anticancer treatment for mammacarcinoma	Radiologists vs radiographic technologists	Agreement between five technologists and radiologists for RECIST 1.1 classification was moderate (k = 0.47 to 0.52) to substantial (k = 0.62 and k = 0.67) Progressive disease classification: strict agreement between technologists and radiologists ranged from substantial to almost perfect (73–97%). Intra-observer agreement among technologists was strong to almost perfect for three readers (κ > 0.78).
Sailer et al	2014	20	Retrospective	CT	RECIST 1.1	Mixed	Treatment for primary tumor	Radiologists vs physician assistants	Interobserver agreement for response category classification was substantial (κ = 0.77; 95% CI: 0.66–0.87). For assessing progressive disease, the radiology physician assistant demonstrated a sensitivity of 100% (95% CI: 61–100%) and specificity of 94% (95% CI: 81–98%).
Hersberger et al	2019	49	Retrospective	MRI	RECIST 1.0 RECIST 1.1	Lung carcinoma	Treatment for primary lung carcinoma	Investigators vs tumor response assessment core (TRAC) web portal vs oncologist vs radiologist	Linearly weighted kappa for concordance: • TRAC vs radiologist: substantial agreement (κ = 0.65; 95% CI: 0.46–0.85; SE: 0.10) • TRAC vs oncologists: moderate agreement (κ = 0.42; 95% CI: 0.20–0.64; SE: 0.11) • Oncologists vs radiologist: fair agreement (κ = 0.34; 95% CI: 0.12–0.55; SE: 0.11)

I. Central assessment versus real-life assessment of tumor response evaluation									
Study	Year	Sample size	Study design	Imaging modality	Response criteria	Primary tumor	Treatment	Comparison	End point
Felsh et al	2017	166	Retrospective	CT	RECIST 1.0	Renal cell carcinoma	Sorafenib or interferon-alpha-2a	Inter-reader variability and the deviation of local from central radiological assessments	Concordance between local and central radiologic review was moderate (κ = 0.53). Progressive disease (PD) rates differed: local: 18.6%, central: 22.5% and only partial overlap between the two. Bland–Altman analysis showed a systematic −7.5% shift in tumor change rates in local vs central assessments, suggesting a bias toward more favorable local results. Discordance impacted time to progression, with a hazard ratio of 1.73 (*p* = 0.0003).
Beaumont et al	2018	179	Retrospective	CT, MRI, PET	RECIST 1.1	Lung carcinoma	Cabazitaxel, topotecan	Local investigators (LI) and blinded independent central review (BICR)	The average number of target lesions per patient was higher in BICR than LI (3.4 vs 2.9; *p* < 0.05), with 18.4% being non-measurable. Discrepancy rates were not linked to non-measurable lesion selection or the use of different imaging scans. But discrepancies were more frequent when LI and BICR selected the same target lesions at baseline.

#### Selection target/measurable lesions

Ten studies compared IOV resulting from radiologists selecting different target or measurable lesions. All concluded that lesion selection is a significant contributor to IOV. However, there is no consensus on the minimum number of lesions required, or on whether selecting more lesions improves agreement. The reasons why different radiologists select different lesions are also not consistently addressed. Additionally, none of the studies clearly differentiate between target lesion selection and measurable lesion selection.

##### Lung carcinoma

Beaumont et al [93] retrospectively compared evaluations by local investigators to blinded independent central reviewers in 179 patients treated with cabazitaxel and topotecan. Local investigators identified fewer target lesions per patient on average compared to the blinded independent central review (2.9 vs 3.4 per patient, respectively), but attributed the majority of discrepancies to finding new lesions, accounting for 53.7% of the differences [30].

##### Other primary tumors

Zhao et al [43] reported that only 33% of 198 target lesions were consistently identified by all three radiologists. Mercier et al [73] took a step further and assessed the variability response among the selected lesions across different organs in 189 patients with colorectal carcinoma treated with bevacizumab or vanucizumab in combination with chemotherapy. While they found clear evidence that shrinkage took longer in vanucizumab than the bevacizumab group, they also identified significant heterogeneity in tumor dynamics across different organs and lesions, which could explain the link between variability in target lesion selection and variability in RECIST categories.

Moskowitz et al [51] conducted a simulation to assess whether the number of lesions measured affects response evaluation and IOV. They found minimal disagreement between response assessments based on five versus ten lesions. However, measuring fewer than five lesions resulted in significant disagreement. Canals-Lambarri et al [11], on the other hand, found that two-to-four target lesions are generally sufficient, with higher ICC in colorectal liver metastases (requiring less lesions) and lower ICC in renal cell (requiring more lesions).

Not specifically on the selection of target lesions, Muenzel et al [28] examined the extent of intra- and inter-variability in target lesion measurement and found that it differed in 6.3% of cases when re-assessed by the same observer and in 12% of cases when re-assessed by a different observer, with the ‘true response’ estimated via consensus.

In mesothelioma, target lesions are replaced by target locations. Armato et al [8] observed systematic bias, with some readers consistently measuring thicker or thinner parts of the tumor compared to others. In metastatic breast cancer, Keil et al [24] reported a significant difference in IOV when comparing cases where the same lesions were selected (76% agreement) compared to cases where different were selected (19% agreement). Krasovitsky et al [25] reported a high intra- and interobserver “reproducibility rate” in selecting target lesions in ovarian carcinoma. More specifically, peritoneal lesions showed the highest selection reproducibility rate (0.76), while lymph nodes exhibited the lowest (0.58). See Table 1B for an overview of all studies.

#### Inter- and intra-observer variability in repeated lesion measurements using the same imaging modality

Oxnard et al [31] prospectively studied the variability of tumor measurements on test-retest CT scans (max 15 min apart) in 30 patients with NSCLC. They found that changes in tumor diameter exceeding 1 to 2 mm are common (33–57% of different magnitude changes) found on immediate reimaging. The median change in tumor measurements found on repeat CT scans performed within 15 min of each other ranged from a 23% shrinkage to a 31% growth, with three patients meeting the cut-offs for RECIST Progressive disease (PD). See Table 1D.

#### Unidimensional diameter

Twenty-three studies investigated IOV induced by 1D diameter. Often these studies compared 1D diameters against 2D cross-sectional areas or 3D volumes. There is no consistent advantage of 3D over 1D or 2D in terms of IOV when using manual measurements only, but 3D is more accurate for irregular lesions and in phantom experiments.

##### One-dimensional versus three-dimensional

In general, 3D measurements were mostly explored with semi-automatic algorithms. To avoid repetition with Section 3.5.1, we report only investigations with manual 3D vs manual 1D measurements, or semi-automatic 3D vs semi-automatic 1D measurements.

Fabel et al [47] prospectively compared manual diameters and semi-automated volumes of 227 enlarged lymph nodes of 47 patients. Using Bland–Altman they found a limit of agreement (LoA) of 5.84 mm for manual 1D measurements, 2.87 mm for manual 3D. Schoot et al [82] found moderate IOV for both diameters (RECIST, κ = 0.59) and bounding-ellipsoid volumes (European Pediatric Soft-Tissue Sarcoma Study Group (EpSGG), κ = 0.57) in 124 sarcoma patients receiving chemotherapy, with a 20% discrepancy in response classification between the two criteria, but evenly balanced between underestimation and overestimation of response. Steger et al [37] reported a 10% IOV (measured in relative standard deviation) for manual diameters, compared to nearly 16% for bounding box volumes of lymph nodes in head and neck carcinoma. The authors further discussed that the actual lymph node volume segmentation “performs a lot better,” but this was not commonly available at the time of the investigation. In terms of actual manual 3D tumor segmentation, Hajkova et al [20] incorporated volume alongside RECIST and mRECIST in a prospective study of transarterial chemoembolization (TACE) patients to evaluate its prognostic value, but did not find a significant difference in terms of interobserver variability (Pearson *r* > 0.94). Only one criterion incorporates volume: the 3D quantitative European Association for the Study of the Liver (qEASL). However, a retrospective study of 57 patients with hepatocellular carcinoma [84] compared mRECIST (1D) with qEASL, where they reported high IOV for mRECIST (max k = 0.43), but did not report it for qEASL.

*One-dimensional versus bidimensional versus three-dimensional:* Zhao et al [43] used a cohort of 29 patients from a clinical trial and mixed models to analyze variability and found higher relative change in the sum of measurements between readers for semi-automatic 2D and semi-automatic 3D measurements (19% and 22% on the sum of measurements, 14% and 12% for the same lesion) compared to semi-automatic 1D measurements (11% and 8% respectively). Villemaire et al [55] found that for spherical-shaped phantoms, all techniques demonstrated high accuracy (no significant difference compared with the ground truth). However, for irregularly shaped phantoms, only volume measurements showed strong agreement with the ground truth, while 1D and 2D were significantly different. They also repeated the analysis in 29 patients but found that all measurements had strong intra- and interobserver agreement (ICC > 0.9).

*One-dimensional versus bidimensional measurements:* Nishino et al [61] compared the best overall response in immune-related response and time to progression between diameters and cross-sectional area in 57 patients, finding high concordance between the two criteria (Weighted Kappa = 0.881), but the number of patients progressing at 6 months was 70% according to cross-sectional area and 81% according to diameters. See Table 1D for an overview of all studies.

#### Radiologist versus non-radiologists

Three papers (3.4%) compared the review of scans by radiologists to those by non-radiologists. Gouel et al [19] examined the reviews of radiologists and radiographic technologists for CT scans in evaluating the response of 90 patients with breast cancer. The agreement rate in RECIST 1.1 classifications between five radiographic technologists and radiologists showed κ = 0.47–0.67. Sailer et al [54] assessed the sensitivity and specificity of CT scan evaluations by radiologists versus radiology physician assistants in 20 patients with mixed tumors. The sensitivity and specificity for radiology physician assistants in assessing PD were 100% (95% CI: 61–100%) and 94% (95% CI: 81–98%), respectively. Hersberger et al [72] compared the assessments of investigators using a tumor response assessment core (TRAC) web portal to the response assessments by oncologists and radiologists when reviewing MRI scans for 47 lung carcinoma patients. The κ value for TRAC versus oncologists was 0.42, and for oncologists versus radiologists, it was 0.34.

Although only a few studies are available, these suggest that non-radiologists can achieve substantial agreement with radiologists. However, we cannot conclude whether their lack of radiological training can impact IOV, either positively or negatively.

#### Radiologist involvement

We identified two retrospective papers (2.3%). Felsh et al [62] examined the interreader variability and deviation of local assessments from central radiological assessments in a group of 170 renal cell carcinoma patients treated with sorafenib or interferon-alpha-2a. The concordance between local and central radiological reviews was κ = 0.53. Central reviewers identified more cases of PD compared to local reviewers (22.5% vs 18.6%). A systemic shift was observed in tumor change rates, with local assessments showing a −7.5% deviation compared to central assessments, indicating a tendency toward more favorable results in local reviews.

Beaumont et al [93] compared the assessments of local investigators with those of a blinded independent central review in 179 lung carcinoma patients treated with cabazitaxel and topotecan. The average number of target lesions identified by local investigators was lower than that identified by the blinded independent central review (2.9 vs 3.4 per patient). Interestingly, when local investigators and the blinded independent central review selected the same target lesions at baseline, more discrepancies occurred in the subsequent response evaluations. Most of the discrepancies were explained by the difference in detecting new lesions.

#### Type of criteria

Nineteen studies compared specialized response criteria with standard RECIST. With the exception of hepatocellular carcinoma, which is discussed separately below, all studies focused on criteria that either do not rely on 1D measurements (e.g., WHO, EpSSG) or do not use target lesions (e.g., mRECIST for mesothelioma). These are summarized in the relevant sections above. Overall, there is no clear consensus that specialized criteria consistently reduce IOV compared to standard RECIST.

##### Hepatocellular carcinoma

Sato et al [33] found higher response rates with mRECIST in 21 patients receiving TACE compared to RECIST (CR: 56.9% vs 9.2%; PR: 79.7% vs 43.1%) and lower intra- and interobserver variability (k = 0.90, 0.82 vs 0.64, 0.63, respectively). Seyal et al [34] confirmed in 103 patients that mRECIST showed higher response rates (40.8% vs 3.9%) earlier in the treatment. They reported intra- and IOV for mRECIST (κ = 0.70 and 0.56), but did not report it for RECIST. They found, however, a low agreement between the two criteria (κ = 0.18). Tovoli et al [83], on the other hand, who also included Response Evaluation Criteria in Cancer of the Liver (RECICL) in their analysis of 77 sorafenib-treated patients, found similar levels of IOV in each of the three methods (k = 0.70–0.75), which were significantly higher among expert readers (k = 0.81–0.87).

Kim et al [49] reported a comparable concordance coefficient in EASL and mRECIST and measurements for both absolute (CCC = 0.99) and relative change (%, CCC = 0.87, 0.88) in 133 patients undergoing chemoembolization, and excellent agreement between response classes of EASL and mRECIST (k > 0.96). King et al [84] included Liver Imaging Reporting and Data System (LI-RADS) and qEASL (using a bounding box volume) in 57 patients who underwent radioembolization and found IOV of κ = 0.43 for mRECIST and κ = 0.48 for LI-RADS. They did not report IOV for qEASL, but they noticed significant correlations between qEASL and the other methods.

Ghosn et al [86] compared the prognostic value in 31 patients using RECIST 1.1, mRECIST, vRECIST, EASL, qEASL, and LIRADS, finding similar hazard ratios among them (HR = 0.22–0.30) but reported RECIST 1.1 to have the highest inter-reader agreement (k = 0.77 vs k < 0.64 of the other methods). Pirasteh et al [89] observed moderate IOV in 82 patients between LIRADS Response to Treatment (LRTR) (κ = 0.42–0.57) and mRECIST (κ = 0.62–0.66). See Table 1E for an overview of all studies.

#### Different imaging techniques

Seven studies investigate the role of imaging techniques on IOV. There is a consensus that the modality of choice can influence the lesion estimate. Some studies show that MRI offers lower IOV than CT.

Plathow et al [85] retrospectively compared the mRECIST for pleural mesothelioma and RECIST using MRI and CT scans in a cohort of 50 mesothelioma patients treated with chemotherapy and observed that MRI had lower interobserver variability than CT (k = 0.7–1.0, vs 0.6–1.0, respectively). Similarly, Moalla et al [81] found that liver MRI measurement had lower interobserver relative variation (IRV) compared to CT (IRV < 20% in 97% vs 81%) in 53 patients with neuro-endocrine tumors who were either treatment-naïve or treated with somatostatin analogues. Yang et al [92] compared tumor sizes assessed by CT and MRI with measurements of resected pathological specimens from 114 patients diagnosed with pancreatic ductal adenocarcinoma. They found that both CT and MRI had good agreement with pathological size (ICC > 0.6) but were estimated consistently smaller on imaging when the tumor exceeded 30 mm in size. The mean difference was larger with MRI than with CT (5.8 mm vs 4.3 mm). Luersen et al [70] conducted a retrospective study on intra- and interobserver variability in measuring the size of liver metastases in 30 patients with carcinoid tumors using various MR sequences (T1-weighted, T2-weighted, diffusion-weighted imaging (DWI), pre- and post-Gadolinium (Gd)). They found that measurements with hepatobiliary phase (HBP)-Gd exhibited the least IOV. Additionally, lesion sizes measured on DWI were notably larger compared to measurements from other sequences. Huh et al [21] retrospectively compared the different phases of CT scans (pre-contrast, arterial phase, and portal venous phase) in 44 patients with neuro-endocrine liver metastases. In this study, two blinded readers measured the longest diameters of target lesions on the three phases twice. They observed improved inter- and intra-observer agreement (Bland–Altman) in size measurements on pre-contrast scans compared to arterial phase and portal venous phase scans. See Table 1F for an overview of all studies.

#### Radiological follow-up

We could not find any published studies comparing different time periods for response evaluation.

## Discussion

Our review showed that IOV in RECIST assessments remains a major challenge in oncology, affecting different tumor types, imaging modalities, and response criteria. While quantitative comparisons across studies are limited due to heterogeneity in metrics, measurement methods, and study designs, Fig. 1 illustrates an overview of potential factors contributing to IOV.IOV may influence tumor response evaluation and clinical decisions, although the magnitude of this impact remains uncertain [94, 95]. However, the clinical impact of IOV may be limited. Mandrekar et al showed that using a 2- or 3-category response system, or varying RECIST cut points for PR and PD, did not meaningfully alter the prediction of overall survival across multiple tumor types {Mandrekar, 2014 #1910}. These findings suggest that, although IOV introduces variability in tumor measurements, its effect on patient outcomes may be smaller than other factors, such as lesion selection, measurement method, or imaging quality. There is a consensus that the method of lesion measurement (manual versus semi-automatic, 1D compared to 3D), the specific lesions evaluated (target lesion or measurable selections), and the quality and resolution of the image (MRI vs CT, contrast vs non-contrast) significantly contribute to IOV. However, there is no consensus on how radiologists’ experience, their involvement in the trial, modifications of response criteria for specific tumor types influence, or differences in follow-up schedules on IOV.

In this review, we could only determine whether there is consensus on a given factor’s association with IOV. For factors where no consensus was found, a meta-analysis would be needed to draw stronger conclusions, but this would require a comparable study design, which is currently lacking.

Statistical methods are often misused. For response categories, the Kappa coefficient should be used [96]. For continuous values, the intraclass correlation coefficient (ICC) is preferred [97]. The coefficient of variation (CV) can help quantify the relative variability of continuous measurements, but it relies on the standard deviation; therefore, it is recommended only when a large number of readers is involved [98]. Percentage agreement, Bland–Altman plots, correlation coefficients, and statistical testing of mean differences are, generally speaking, inappropriate to quantify IOV. Although statistical methods to assess agreement are widely used, their application and interpretation are not always optimal. For example, a previous study reported Pearson correlation coefficients to quantify agreement between readers. However, correlation measures the strength of association rather than agreement, and can therefore be high even in the presence of systematic differences between observers {Janse, 2021 #1928}. For instance, two readers may show a strong linear relationship in their measurements while consistently differing in absolute values, which would not be detected by correlation analysis alone. In addition, correlation coefficients may be inflated when measurements span a wide range, as is common in tumor size assessments.

Another study reported intraclass correlation coefficients (ICC) without clearly specifying the model or type used, which may affect interpretability and comparability across studies {Koo, 2016 #1929}. Similarly, while kappa statistics and percentage agreement were frequently used for categorical response assessment, their interpretation can be influenced by category prevalence and does not provide insight into the magnitude or clinical relevance of disagreement {O’Leary, 2014 #1930}.

These examples illustrate the importance of selecting appropriate statistical methods for agreement analysis. Approaches such as Bland–Altman analysis and clearly specified ICC models are generally more informative for assessing agreement in continuous measurements.

Statistical methods can also be misinterpreted. For example, Kappa can be the three classes of response (PD, PR, SD) or dichotomized as (PD, SD) vs (PR), or (PD) vs (SD, PR). It can be applied for two raters (Cohen) or three or more (Fleiss). Moreover, Kappa can be weighted if we want to account for a natural order between the classes, e.g., PR before SD, before PD. These are all reported as Kappa, but their values are not directly comparable, since each metric of Kappa is based on different assumptions or computations.

Percentage agreement and the kappa coefficient are the most commonly used metrics for comparing response categories between readers. For continuous measurements, frequently reported metrics include Bland–Altman analysis, correlation coefficients, intraclass correlation coefficients (ICC), and coefficients of variation (CV). However, these metrics are not interchangeable and should be interpreted with caution. For example, correlation coefficients quantify the strength of a linear relationship rather than agreement, and can therefore be high even when there is systematic disagreement between observers. In contrast, methods such as Bland–Altman analysis are more appropriate for assessing agreement, as they evaluate both bias and limits of agreement. A comprehensive evaluation of statistical methodology is beyond the scope of this review, but appropriate selection and interpretation of these metrics remain essential.

Some interpretations are misleading. Commonly used qualitative labels for Kappa values (e.g., “excellent,” “good,” “fair”) can be misleading, as they are not universally applicable and depend on the specific type of Kappa used. These thresholds were first proposed by Landis and Koch (1977), who themselves acknowledged them as arbitrary and intended only as guidelines for a specific example, not as general standards [97]. Later, it was pointed out how this could be more harmful than useful [99]. Even worse, in some cases, the same interpretations have been implemented for other IOV metrics, most commonly the ICC, giving a false sense of “inter-metrics comparability.” A key limitation of current IOV metrics is their lack of patient-centered interpretability. For example, widely used metrics like positive predictive value (PPV) directly inform clinical decision-making by indicating the probability of a false positive in a patient testing positive—information that can guide treatment, retesting, or patient counseling. In contrast, Kappa values offer limited clinical insight, as they do not translate easily into a patient’s risk of diagnostic disagreement or misclassification. Bakeman et al [100], in examining the impact of class number and imbalance on Kappa, linked it to more intuitive accuracy metrics. For instance, a Kappa of 0.6 in a balanced two-class scenario corresponds to an accuracy of 0.8, implying disagreement in 2 out of 10 cases. Since only one reader can be correct in any disagreement, this translates to an estimated 1 in 10 patients being misclassified. A comprehensive review of IOV metric interpretation is beyond the scope of this paper, but we believe such an evaluation is both necessary and overdue. Additionally, we did not perform a formal risk-of-bias or quality assessment of the included studies due to the wide variety of tumor types, imaging modalities, and study designs. Therefore, our review is descriptive, and readers should interpret the findings accordingly.

In this review, we analyzed a large body of literature, enabling a comprehensive summary of IOV in RECIST assessments and its contributing factors. However, our search may not have captured all relevant studies, particularly those evaluating 1D measurement reproducibility without explicitly referencing RECIST. Additionally, we limited our scope to studies published after the release of RECIST 1.1 (December 2008), though findings from RECIST 1.0 may also be pertinent. As new response criteria emerge, especially with the integration of AI, we recognize the need for standardized protocols to evaluate IOV moving forward. In our review, we analyzed a large number of studies, allowing us to comprehensively summarize the current literature on RECIST IOV and the factors that may contribute to it. However, the query itself might not have been comprehensive of all studies that could have reported relevant results. These, for example, could be studies that investigated the reproducibility of 1D diameter but did not necessarily mention RECIST. We are aware that, especially with the introduction of AI, novel criteria are being developed that can overcome some of these factors. We hope that at that time we will have a standardized protocol to assess IOV.

## Conclusion

In conclusion, our review highlights that interobserver variability remains an important consideration in RECIST assessments. Key contributors to IOV include manual measurements, lesion selection, and differences in imaging quality and methodology. Several factors remain understudied, and standardized guidelines for evaluating IOV are lacking. Future RECIST updates could consider semi-automatic and volumetric measurement approaches as potential tools to reduce IOV. However, given the heterogeneous and limited evidence base, further studies are needed to determine their clinical utility and reproducibility across different tumor types and imaging modalities.

## Supplementary information

ELECTRONIC SUPPLEMENTARY MATERIAL

## Data Availability

All data analyzed in this systematic review are derived from published literature and publicly available sources.

## Abbreviations

1D: One-dimensional
2D: Two-dimensional
3D: Three-dimensional
ANCOVA: Analysis of covariance
CI: Confidence interval
CR: Complete response
CT: Computed tomography
CV: Coefficient of variation
DOI: Digital Object Identifier
DWI: Diffusion-weighted imaging
EpSGG: European Pediatric Soft-Tissue Sarcoma Study Group
Gd: Gadolinium
HR: Hazard ratio
ICC: Intraclass correlation
IOV: Inter-/intra-observer variability
IQR: Interquartile range
K: Kappa
LI-RADS: Liver Imaging Reporting and Data System
MRI: Magnetic resonance imaging
NET: Neuro-endocrine tumors
NSCLC: Non-small cell lung cancer
OS: Overall survival
PD: Progressive disease
PET: Positron emission tomography
PFS: Progression-free survival
PR: Partial response
PRISMA: Preferred Reporting Items for Systematic Reviews and Meta-Analyses
PVP: Portal venous phase
qEASL: Quantitative European Association for the Study of the Liver
RECICL: Response Evaluation Criteria in Cancer of the Liver
RECIST: Response Evaluation Criteria In Solid Tumors
SD: Standard deviation
T1: T1-weighted imaging
T2: T2-weighted imaging
TACE: Transarterial chemoembolization
TFS: Tumor-free survival
TRAC: Tumor response assessment core
WHO: World Health Organization

## References

[1] Eisenhauer EA, Therasse P, Bogaerts J et al (2009) New response evaluation criteria in solid tumours: revised RECIST guideline (version 1.1). Eur J Cancer 45:228–247. 10.1016/j.ejca.2008.10.026 19097774 10.1016/j.ejca.2008.10.026

[2] Therasse P, Arbuck SG, Eisenhauer EA et al (2000) New guidelines to evaluate the response to treatment in solid tumors. European Organization for Research and Treatment of Cancer, National Cancer Institute of the United States, National Cancer Institute of Canada. J Natl Cancer Inst 92:205–216. 10.1093/jnci/92.3.205 10655437 10.1093/jnci/92.3.205

[3] Sharma MR, Maitland ML, Ratain MJ (2012) RECIST: no longer the sharpest tool in the oncology clinical trials toolbox-point. Cancer Res 72:5145–5149. 10.1158/0008-5472.CAN-12-0058 22952219 10.1158/0008-5472.CAN-12-0058

[4] Villaruz LC, Socinski MA (2013) The clinical viewpoint: definitions, limitations of RECIST, practical considerations of measurement. Clin Cancer Res 19:2629–2636. 10.1158/1078-0432.CCR-12-2935 23669423 10.1158/1078-0432.CCR-12-2935 PMC4844002

[5] Page MJ, McKenzie JE, Bossuyt PM et al (2021) The PRISMA 2020 statement: an updated guideline for reporting systematic reviews. J Clin Epidemiol 134:178–189 33789819 10.1016/j.jclinepi.2021.03.001

[6] Litière S, Isaac G, De Vries E et al (2019) RECIST 1.1 for response evaluation apply not only to chemotherapy-treated patients but also to targeted cancer agents: a pooled database analysis. J Clin Oncol 37:1102–1110. 10.1200/JCO.18.01100 30860949 10.1200/JCO.18.01100 PMC6494357

[7] Bellomi M, De Piano F, Ancona E et al (2017) Evaluation of inter-observer variability according to RECIST 1.1 and its influence on response classification in CT measurement of liver metastases. Eur J Radiol 95:96–101. 10.1016/j.ejrad.2017.08.001 28987705 10.1016/j.ejrad.2017.08.001

[8] Armato SG 3rd, Nowak AK, Francis RJ, Kocherginsky M, Byrne MJ (2014) Observer variability in mesothelioma tumor thickness measurements: defining minimally measurable lesions. J Thorac Oncol 9:1187–1194. 10.1097/JTO.0000000000000211 25157772 10.1097/JTO.0000000000000211

[9] Awad J, Owrangi A, Villemaire L, O'Riordan E, Parraga G, Fenster A (2012) Three-dimensional lung tumor segmentation from x-ray computed tomography using sparse field active models. Med Phys 39:851–865. 10.1118/1.3676687 22320795 10.1118/1.3676687

[10] Bendtsen C, Kietzmann M, Korn R, Mozley PD, Schmidt G, Binnig G (2011) X-ray computed tomography: semiautomated volumetric analysis of late-stage lung tumors as a basis for response assessments. Int J Biomed Imaging 2011:361589. 10.1155/2011/361589 21747819 10.1155/2011/361589 PMC3124287

[11] Canals-Lambarri M, Canals-Cifuentes A, Barros-Rocco A, Barros-Nelson P, Mahave-Caceres M, Salman-Boghikian P (2015) Assessment of the usefulness of the quantitative methods for the response evaluation of solid tumors: analysis using four cancer types. Rev Invest Clin 67:182–190 26202742

[12] Choi MH, Park GE, Oh SN et al (2018) Reproducibility of mRECIST in measurement and response assessment for hepatocellular carcinoma treated by transarterial chemoembolization. Acad Radiol 25:1363–1373. 10.1016/j.acra.2018.02.013 29555570 10.1016/j.acra.2018.02.013

[13] Colombi D, Manna C, Montermini I et al (2015) Semiautomatic analysis on computed tomography in locally advanced or metastatic non-small cell lung cancer: reproducibility and prognostic significance of unidimensional and 3-dimensional measurements. J Thorac Imaging 30:290–299. 10.1097/RTI.0000000000000145 25837590 10.1097/RTI.0000000000000145

[14] Cornelis FH, Martin M, Saut O et al (2017) Precision of manual two-dimensional segmentations of lung and liver metastases and its impact on tumour response assessment using RECIST 1.1. Eur Radiol Exp 1:16. 10.1186/s41747-017-0015-4 29708185 10.1186/s41747-017-0015-4 PMC5909353

[15] Dicken V, Bornemann L, Moltz JH, Peitgen HO, Zaim S, Scheuring U (2015) Comparison of volumetric and linear serial CT assessments of lung metastases in renal cell carcinoma patients in a clinical phase IIB study. Acad Radiol 22:619–625. 10.1016/j.acra.2014.12.018 25778472 10.1016/j.acra.2014.12.018

[16] Fabel M, Bolte H, von Tengg-Kobligk H et al (2011) Semi-automated volumetric analysis of lymph node metastases during follow-up—initial results. Eur Radiol 21:683–692. 10.1007/s00330-010-1966-5 20953870 10.1007/s00330-010-1966-5

[17] Fabel M, Wulff A, Heckel F et al (2012) Clinical lymph node staging—influence of slice thickness and reconstruction kernel on volumetry and RECIST measurements. Eur J Radiol 81:3124–3130. 10.1016/j.ejrad.2012.03.008 22464844 10.1016/j.ejrad.2012.03.008

[18] Folio LR, Sandouk A, Huang J, Solomon JM, Apolo AB (2013) Consistency and efficiency of CT analysis of metastatic disease: semiautomated lesion management application within a PACS. AJR Am J Roentgenol 201:618–625. 10.2214/AJR.12.10136 23971455 10.2214/AJR.12.10136 PMC6771287

[19] Gouel P, Callonnec F, Levêque É et al (2023) Evaluation of the capability and reproducibility of RECIST 1.1. measurements by technologists in breast cancer follow-up: a pilot study. Sci Rep 13:9148. 10.1038/s41598-023-36315-w 37277412 10.1038/s41598-023-36315-w PMC10241950

[20] Hajkova M, Andrasina T, Ovesna P et al (2022) Volumetric analysis of hepatocellular carcinoma after transarterial chemoembolization and its impact on overall survival. Vivo 36:2332–2341. 10.21873/invivo.12964 10.21873/invivo.12964 PMC9463903 36099102

[21] Huh J, Park J, Kim KW et al (2018) Optimal phase of dynamic computed tomography for reliable size measurement of metastatic neuroendocrine tumors of the liver: comparison between pre- and post-contrast phases. Korean J Radiol 19:1066–1076. 10.3348/kjr.2018.19.6.1066 30386138 10.3348/kjr.2018.19.6.1066 PMC6201971

[22] Iannessi A, Beaumont H (2023) Breaking down the RECIST 1.1 double read variability in lung trials: what do baseline assessments tell us? Front Oncol 13:988784. 10.3389/fonc.2023.988784 37007064 10.3389/fonc.2023.988784 PMC10060958

[23] Karmakar A, Kumtakar A, Sehgal H, Kumar S, Kalyanpur A (2019) Interobserver variation in Response Evaluation Criteria in Solid Tumors 1.1. Acad Radiol 26:489–501. 10.1016/j.acra.2018.05.017 29934024 10.1016/j.acra.2018.05.017

[24] Keil S, Barabasch A, Dirrichs T et al (2014) Target lesion selection: an important factor causing variability of response classification in the Response Evaluation Criteria for Solid Tumors 1.1. Invest Radiol 49:509–517. 10.1097/RLI.0000000000000048 24651664 10.1097/RLI.0000000000000048

[25] Krasovitsky M, Lee YC, Sim HW et al (2022) Interobserver and intraobserver variability of RECIST assessment in ovarian cancer. Int J Gynecol Cancer 32:656–661. 10.1136/ijgc-2021-003319 35379690 10.1136/ijgc-2021-003319

[26] Li H, Shen J, Shou J et al (2021) Exploring the interobserver agreement in computer-aided radiologic tumor measurement and evaluation of tumor response. Front Oncol 11:691638. 10.3389/fonc.2021.691638 35174064 10.3389/fonc.2021.691638 PMC8841678

[27] Mozley PD, Bendtsen C, Zhao B et al (2012) Measurement of tumor volumes improves RECIST-based response assessments in advanced lung cancer. Transl Oncol 5:19–25. 10.1593/tlo.11232 22348172 10.1593/tlo.11232 PMC3281412

[28] Muenzel D, Engels HP, Bruegel M, Kehl V, Rummeny EJ, Metz S (2012) Intra- and inter-observer variability in measurement of target lesions: implication on response evaluation according to RECIST 1.1. Radiol Oncol 46:8–18. 10.2478/v10019-012-0009-z 22933974 10.2478/v10019-012-0009-z PMC3423763

[29] Nishino M, Gargano M, Suda M, Ramaiya NH, Hodi FS (2014) Optimizing immune-related tumor response assessment: does reducing the number of lesions impact response assessment in melanoma patients treated with ipilimumab? J Immunother Cancer 2:17. 10.1186/2051-1426-2-17 24991412 10.1186/2051-1426-2-17 PMC4077549

[30] Oubel E, Bonnard E, Sueoka-Aragane N et al (2015) Volume-based response evaluation with consensual lesion selection: a pilot study by using cloud solutions and comparison to RECIST 1.1. Acad Radiol 22:217–225. 10.1016/j.acra.2014.09.008 25488429 10.1016/j.acra.2014.09.008

[31] Oxnard GR, Zhao B, Sima CS et al (2011) Variability of lung tumor measurements on repeat computed tomography scans taken within 15 min. J Clin Oncol 29:3114–3119. 10.1200/JCO.2010.33.7071 21730273 10.1200/JCO.2010.33.7071 PMC3157977

[32] Rothe JH, Grieser C, Lehmkuhl L et al (2013) Size determination and response assessment of liver metastases with computed tomography-comparison of RECIST and volumetric algorithms. Eur J Radiol 82:1831–1839. 10.1016/j.ejrad.2012.05.018 22717124 10.1016/j.ejrad.2012.05.018

[33] Sato Y, Watanabe H, Sone M et al (2013) Tumor response evaluation criteria for HCC (hepatocellular carcinoma) treated using TACE (transcatheter arterial chemoembolization): RECIST (response evaluation criteria in solid tumors) version 1.1 and mRECIST (modified RECIST): JIVROSG-0602. Ups J Med Sci 118:16–22. 10.3109/03009734.2012.729104 23167460 10.3109/03009734.2012.729104 PMC3572665

[34] Seyal AR, Gonzalez-Guindalini FD, Arslanoglu A et al (2015) Reproducibility of mRECIST in assessing response to transarterial radioembolization therapy in hepatocellular carcinoma. Hepatology 62:1111–1121. 10.1002/hep.27915 25999236 10.1002/hep.27915

[35] Skougaard K, McCullagh MJ, Nielsen D, Hendel HW, Jensen BV, Johannesen HH (2012) Observer variability in a phase II trial—assessing consistency in RECIST application. Acta Oncol 51:774–780. 10.3109/0284186X.2012.667149 22432439 10.3109/0284186X.2012.667149

[36] Sohns C, Mangelsdorf J, Sossalla S, Konietschke F, Obenauer S (2010) Measurement of response of pulmonal tumors in 64-slice MDCT. Acta Radiol 51:512–521. 10.3109/02841851003674520 20540683 10.3109/02841851003674520

[37] Steger S, Franco F, Sverzellati N, Chiari G, Colomer R (2011) 3D assessment of lymph nodes vs. RECIST 1.1. Acad Radiol 18:391–394. 10.1016/j.acra.2010.11.010 21216161 10.1016/j.acra.2010.11.010

[38] Suzuki C, Torkzad MR, Jacobsson H et al (2010) Interobserver and intraobserver variability in the response evaluation of cancer therapy according to RECIST and WHO-criteria. Acta Oncol 49:509–514. 10.3109/02841861003705794 20397778 10.3109/02841861003705794

[39] van Kessel CS, van Leeuwen MS, Witteveen PO, Kwee TC, Verkooijen HM, van Hillegersberg R (2012) Semi-automatic software increases CT measurement accuracy but not response classification of colorectal liver metastases after chemotherapy. Eur J Radiol 81:2543–2549. 10.1016/j.ejrad.2011.12.026 22264447 10.1016/j.ejrad.2011.12.026

[40] Wesdorp NJ, Kemna R, Bolhuis K et al (2022) Interobserver variability in CT-based morphologic tumor response assessment of colorectal liver metastases. Radiol Imaging Cancer 4:210105. 10.1148/rycan.210105 10.1148/rycan.210105 PMC9152692 35522139

[41] Woo M, Heo M, Devane AM, Lowe SC, Gimbel RW (2020) Retrospective comparison of approaches to evaluating inter-observer variability in CT tumour measurements in an academic health centre. BMJ Open 10:e040096. 10.1136/bmjopen-2020-040096 33191265 10.1136/bmjopen-2020-040096 PMC7668356

[42] Wulff AM, Fabel M, Freitag-Wolf S et al (2013) Volumetric response classification in metastatic solid tumors on MSCT: initial results in a whole-body setting. Eur J Radiol 82:e567–e573. 10.1016/j.ejrad.2013.05.030 23827800 10.1016/j.ejrad.2013.05.030

[43] Zhao B, Lee SM, Lee HJ et al (2014) Variability in assessing treatment response: metastatic colorectal cancer as a paradigm. Clin Cancer Res 20:3560–3568. 10.1158/1078-0432.CCR-14-0245 24780294 10.1158/1078-0432.CCR-14-0245 PMC4337392

[44] Zheng B, Shin JH, Li H, Chen Y, Guo Y, Wang M (2021) Comparison of radiological tumor response based on iRECIST and RECIST 1.1 in metastatic clear-cell renal cell carcinoma patients treated with programmed cell death-1 inhibitor therapy. Korean J Radiol 22:366–375. 10.3348/kjr.2020.0404 33289356 10.3348/kjr.2020.0404 PMC7909853

[45] Zimmermann M, Kuhl C, Engelke H, Bettermann G, Keil S (2021) Volumetric measurements of target lesions: does it improve inter-reader variability for oncological response assessment according to RECIST 1.1 guidelines compared to standard unidimensional measurements? Pol J Radiol 86:e594–e600. 10.5114/pjr.2021.111048 34876940 10.5114/pjr.2021.111048 PMC8634421

[46] Zimmermann M, Kuhl CK, Engelke H, Bettermann G, Keil S (2021) CT-based whole-body tumor volumetry versus RECIST 1.1: feasibility and implications for inter-reader variability. Eur J Radiol 135:109514. 10.1016/j.ejrad.2020.109514 33401109 10.1016/j.ejrad.2020.109514

[47] Fabel M, von Tengg-Kobligk H, Giesel FL et al (2008) Semi-automated volumetric analysis of lymph node metastases in patients with malignant melanoma stage III/IV—a feasibility study. Eur Radiol 18:1114–1122. 10.1007/s00330-008-0866-4 18274757 10.1007/s00330-008-0866-4

[48] Jiang B, Zhou D, Sun Y, Wang J (2017) Systematic analysis of measurement variability in lung cancer with multidetector computed tomography. Ann Thorac Med 12:95–100. 10.4103/1817-1737.203750 28469719 10.4103/1817-1737.203750 PMC5399697

[49] Kim BK, Kim KA, Kim MJ et al (2015) Inter-observer variability of response evaluation criteria for hepatocellular carcinoma treated with chemoembolization. Dig Liver Dis 47:682–688. 10.1016/j.dld.2015.04.004 25977216 10.1016/j.dld.2015.04.004

[50] Krajewski KM, Nishino M, Franchetti Y, Ramaiya NH, Van den Abbeele AD, Choueiri TK (2014) Intraobserver and interobserver variability in computed tomography size and attenuation measurements in patients with renal cell carcinoma receiving antiangiogenic therapy: implications for alternative response criteria. Cancer 120:711–721. 10.1002/cncr.28493 24264883 10.1002/cncr.28493 PMC4031652

[51] Moskowitz CS, Jia X, Schwartz LH, Gonen M (2009) A simulation study to evaluate the impact of the number of lesions measured on response assessment. Eur J Cancer 45:300–310. 10.1016/j.ejca.2008.11.010 19095439 10.1016/j.ejca.2008.11.010 PMC2652848

[52] Öztürk Ç, Velleman T, Bongaerts AH et al (2017) Assessment of volumetric versus manual measurement in disseminated testicular cancer; no difference in assessment between non-radiologists and genitourinary radiologist. PLoS One 12:e0168977. 10.1371/journal.pone.0168977 28081195 10.1371/journal.pone.0168977 PMC5230761

[53] René A, Aufort S, Mohamed S et al (2014) How using dedicated software can improve RECIST readings. Informatics 1:160–173. 10.3390/informatics1020160

[54] Sailer AM, Douwes DC, Cappendijk VC et al (2014) RECIST measurements in cancer treatment: is there a role for physician assistants? A pilot study. Cancer Imaging 14:12. 10.1186/1470-7330-14-12 25608556 10.1186/1470-7330-14-12 PMC4331818

[55] Villemaire L, Owrangi AM, Etemad-Rezai R et al (2011) Pulmonary tumor measurements from x-ray computed tomography in one, two, and three dimensions. Acad Radiol 18:1391–1402. 10.1016/j.acra.2011.07.010 21917485 10.1016/j.acra.2011.07.010

[56] Zhao B, Tan Y, Bell DJ et al (2013) Exploring intra- and inter-reader variability in uni-dimensional, bi-dimensional, and volumetric measurements of solid tumors on CT scans reconstructed at different slice intervals. Eur J Radiol 82:959–968. 10.1016/j.ejrad.2013.02.018 23489982 10.1016/j.ejrad.2013.02.018 PMC3823057

[57] Zimmermann M, Kuhl CK, Engelke H, Bettermann G, Keil S (2021) Factors that drive heterogeneity of response-to-treatment of different metastatic deposits within the same patients as measured by RECIST 1.1 analyses. Acad Radiol 28:e235–e239. 10.1016/j.acra.2020.05.029 32616417 10.1016/j.acra.2020.05.029

[58] Hakozaki T, Okuma Y, Hosomi Y, Hishima T (2019) Radiographic assessment of objective responses using the ITMIG-modified criteria in thymic carcinoma. Oncology 97:264–269. 10.1159/000501104 31307031 10.1159/000501104

[59] Li F, Ahmad M, Qayyum F et al (2019) Correlation of patient survival with clinical tumor measurements in malignant pleural mesothelioma. Eur Radiol 29:2981–2988. 10.1007/s00330-018-5887-z 30617480 10.1007/s00330-018-5887-z

[60] Bregar A, Mojtahed A, Kilcoyne A et al (2019) CT prediction of surgical outcome in patients with advanced epithelial ovarian carcinoma undergoing neoadjuvant chemotherapy. Gynecol Oncol 152:568–573. 10.1016/j.ygyno.2018.12.012 30642626 10.1016/j.ygyno.2018.12.012

[61] Nishino M, Giobbie-Hurder A, Gargano M, Suda M, Ramaiya NH, Hodi FS (2013) Developing a common language for tumor response to immunotherapy: immune-related response criteria using unidimensional measurements. Clin Cancer Res 19:3936–3943. 10.1158/1078-0432.CCR-13-0895 23743568 10.1158/1078-0432.CCR-13-0895 PMC3740724

[62] Felsch M, Zaim S, Dicken V, Lehmacher W, Scheuring UJ (2017) Comparison of central and local serial CT assessments of metastatic renal cell carcinoma patients in a clinical phase IIB study. Acta Radiol 58:249–255. 10.1177/0284185116642634 27083205 10.1177/0284185116642634

[63] Lubner MG, Stabo N, Lubner SJ, Del Rio AM, Song C, Pickhardt PJ (2017) Volumetric versus unidimensional measures of metastatic colorectal cancer in assessing disease response. Clin Colorectal Cancer 16:324–333.e321. 10.1016/j.clcc.2017.03.009 28433601 10.1016/j.clcc.2017.03.009

[64] Monsky WL, Kim I, Loh S et al (2010) Semiautomated segmentation for volumetric analysis of intratumoral ethiodol uptake and subsequent tumor necrosis after chemoembolization. AJR Am J Roentgenol 195:1220–1230. 10.2214/AJR.09.3964 20966331 10.2214/AJR.09.3964

[65] Zhao K, Jiang B, Zhang S et al (2022) Measurement accuracy and repeatability of RECIST-defined pulmonary lesions and lymph nodes in ultra-low-dose CT based on deep learning image reconstruction. Cancers. 10.3390/cancers14205016 10.3390/cancers14205016 PMC9599467 36291800

[66] Kanaly CW, Mehta AI, Ding D et al (2014) A novel, reproducible, and objective method for volumetric magnetic resonance imaging assessment of enhancing glioblastoma. J Neurosurg 121:536–542. 10.3171/2014.4.JNS121952 25036205 10.3171/2014.4.JNS121952 PMC4286293

[67] Bauknecht HC, Romano VC, Rogalla P et al (2010) Intra- and interobserver variability of linear and volumetric measurements of brain metastases using contrast-enhanced magnetic resonance imaging. Invest Radiol 45:49–56. 10.1097/RLI.0b013e3181c02ed5 19996757 10.1097/RLI.0b013e3181c02ed5

[68] Karademir I, Ward E, Peng Y et al (2016) Measurements of hepatic metastasis on MR imaging: assessment of interobserver and intersequence variability. Acad Radiol 23:132–143. 10.1016/j.acra.2015.09.002 26548855 10.1016/j.acra.2015.09.002

[69] Lestra T, Kanagaratnam L, Mulé S et al (2018) Measurement variability of liver metastases from neuroendocrine tumors on different magnetic resonance imaging sequences. Diagn Interv Imaging 99:73–81. 10.1016/j.diii.2017.12.009 29339222 10.1016/j.diii.2017.12.009

[70] Luersen GF, Wei W, Tamm EP, Bhosale PR, Szklaruk J (2016) Evaluation of magnetic resonance (MR) biomarkers for assessment of response with response evaluation criteria in solid tumors: comparison of the measurements of neuroendocrine tumor liver metastases (NETLM) with various MR sequences and at multiple phases of contrast administration. J Comput Assist Tomogr 40:717–722. 10.1097/RCT.0000000000000425 27636124 10.1097/RCT.0000000000000425 PMC5027958

[71] Bonekamp D, Bonekamp S, Halappa VG et al (2014) Interobserver agreement of semi-automated and manual measurements of functional MRI metrics of treatment response in hepatocellular carcinoma. Eur J Radiol 83:487–496. 10.1016/j.ejrad.2013.11.016 24387824 10.1016/j.ejrad.2013.11.016 PMC4380187

[72] Hersberger KE, Mendiratta-Lala M, Fischer R et al (2019) Quantitative imaging assessment for clinical trials in oncology. J Natl Compr Canc Netw 17:1505–1511. 10.6004/jnccn.2019.7331 31805530 10.6004/jnccn.2019.7331

[73] Mercier F, Kerioui M, Desmée S, Guedj J, Krieter O, Bruno R (2020) Longitudinal analysis of organ-specific tumor lesion sizes in metastatic colorectal cancer patients receiving first line standard chemotherapy in combination with anti-angiogenic treatment. J Pharmacokinet Pharmacodyn 47:613–625. 10.1007/s10928-020-09714-z 32865652 10.1007/s10928-020-09714-z

[74] Monsky WL, Jin B, Molloy C et al (2012) Semi-automated volumetric quantification of tumor necrosis in soft tissue sarcoma using contrast-enhanced MRI. Anticancer Res 32:4951–4961 23155265 PMC4180491

[75] Shady W, Sotirchos VS, Do RK et al (2016) Surrogate imaging biomarkers of response of colorectal liver metastases after salvage radioembolization using 90Y-loaded resin microspheres. AJR Am J Roentgenol 207:661–670. 10.2214/AJR.15.15202 27384594 10.2214/AJR.15.15202 PMC5675077

[76] Budjan J, Sauter EA, Morelli JN et al (2016) Semi-automatic volumetric measurement of treatment response in hepatocellular carcinoma after trans-arterial chemoembolization. Anticancer Res 36:4353–4358 27466556

[77] Khokher S, Qureshi MU, Chaudhry NA (2012) Comparison of WHO and RECIST criteria for evaluation of clinical response to chemotherapy in patients with advanced breast cancer. Asian Pac J Cancer Prev 13:3213–3218. 10.7314/apjcp.2012.13.7.3213 22994736 10.7314/apjcp.2012.13.7.3213

[78] Biederer J, Dinkel J, Remmert G et al (2009) 4D-Imaging of the lung: reproducibility of lesion size and displacement on helical CT, MRI, and cone beam CT in a ventilated ex vivo system. Int J Radiat Oncol Biol Phys 73:919–926. 10.1016/j.ijrobp.2008.09.014 19215826 10.1016/j.ijrobp.2008.09.014

[79] Dinkel J, Khalilzadeh O, Hintze C et al (2013) Inter-observer reproducibility of semi-automatic tumor diameter measurement and volumetric analysis in patients with lung cancer. Lung Cancer 82:76–82. 10.1016/j.lungcan.2013.07.006 23932487 10.1016/j.lungcan.2013.07.006

[80] Jeon MY, Lee HW, Kim BK et al (2018) Reproducibility of European Association for the Study of the Liver criteria and modified Response Evaluation Criteria in Solid Tumors in patients treated with sorafenib. Liver Int 38:1655–1663. 10.1111/liv.13731 29495116 10.1111/liv.13731

[81] Moalla S, Arfi Rouche J, Foulon S et al (2017) Are we reproducible in measurement of NET liver metastasis? Dig Liver Dis 49:1121–1127. 10.1016/j.dld.2017.05.015 28844707 10.1016/j.dld.2017.05.015

[82] Schoot RA, McHugh K, van Rijn RR et al (2013) Response assessment in pediatric rhabdomyosarcoma: can response evaluation criteria in solid tumors replace three-dimensional volume assessments? Radiology 269:870–878. 10.1148/radiol.13122607 23985275 10.1148/radiol.13122607

[83] Tovoli F, Renzulli M, Negrini G et al (2018) Inter-operator variability and source of errors in tumour response assessment for hepatocellular carcinoma treated with sorafenib. Eur Radiol 28:3611–3620. 10.1007/s00330-018-5393-3 29633000 10.1007/s00330-018-5393-3

[84] King MJ, Tong A, Dane B, Huang C, Zhan C, Shanbhogue K (2020) Response assessment of hepatocellular carcinoma treated with yttrium-90 radioembolization: inter-reader variability, comparison with 3D quantitative approach, and role in the prediction of clinical outcomes. Eur J Radiol 133:109351. 10.1016/j.ejrad.2020.109351 33096408 10.1016/j.ejrad.2020.109351

[85] Plathow C, Klopp M, Thieke C et al (2008) Therapy response in malignant pleural mesothelioma—role of MRI using RECIST, modified RECIST and volumetric approaches in comparison with CT. Eur Radiol 18:1635–1643. 10.1007/s00330-008-0918-9 18369634 10.1007/s00330-008-0918-9

[86] Ghosn M, Derbel H, Kharrat R et al (2021) Prediction of overall survival in patients with hepatocellular carcinoma treated with Y-90 radioembolization by imaging response criteria. Diagn Interv Imaging 102:35–44. 10.1016/j.diii.2020.09.004 33012693 10.1016/j.diii.2020.09.004

[87] Huang RY, Unadkat P, Bi WL et al (2019) Response assessment of meningioma: 1D, 2D, and volumetric criteria for treatment response and tumor progression. Neuro Oncol 21:234–241. 10.1093/neuonc/noy126 30085283 10.1093/neuonc/noy126 PMC6374755

[88] Mattonen SA, Palma DA, Haasbeek CJ, Senan S, Ward AD (2013) Distinguishing radiation fibrosis from tumour recurrence after stereotactic ablative radiotherapy (SABR) for lung cancer: a quantitative analysis of CT density changes. Acta Oncol 52:910–918. 10.3109/0284186X.2012.731525 23106174 10.3109/0284186X.2012.731525

[89] Pirasteh A, Sorra EA, Marquez H et al (2021) LI-RADS treatment response algorithm after first-line DEB-TACE: reproducibility and prognostic value at initial post-treatment CT/MRI. Abdom Radiol 46:3708–3716. 10.1007/s00261-021-03043-6 10.1007/s00261-021-03043-6 33755735

[90] Schvartsman G, Wagner MJ, Amini B et al (2017) Treatment patterns, efficacy and toxicity of regorafenib in gastrointestinal stromal tumour patients. Sci Rep 7:9519. 10.1038/s41598-017-09132-1 28842575 10.1038/s41598-017-09132-1 PMC5573380

[91] Staal FC, Beets-Tan RG, Heeres BC et al (2021) Magnetic resonance assessment of sinusoidal obstruction syndrome after neoadjuvant chemotherapy for colorectal liver metastases is not reproducible. Acta Radiol 62:1133–1141. 10.1177/0284185120957988 32972213 10.1177/0284185120957988

[92] Ma C, Yang P, Li J, Bian Y, Wang L, Lu J (2020) Pancreatic adenocarcinoma: variability in measurements of tumor size among computed tomography, magnetic resonance imaging, and pathologic specimens. Abdom Radiol 45:782–788. 10.1007/s00261-019-02125-w 10.1007/s00261-019-02125-w 31292672

[93] Beaumont H, Evans TL, Klifa C et al (2018) Discrepancies of assessments in a RECIST 1.1 phase II clinical trial—association between adjudication rate and variability in images and tumors selection. Cancer Imaging 18:50. 10.1186/s40644-018-0186-0 30537991 10.1186/s40644-018-0186-0 PMC6288919

[94] Baey C, Le Deley MC (2011) Effect of a misspecification of response rates on type I and type II errors, in a phase II Simon design. Eur J Cancer 47:1647–1652. 10.1016/j.ejca.2011.03.013 21493059 10.1016/j.ejca.2011.03.013

[95] Buyse M, Squifflet P, Coart E, Quinaux E, Punt CJ, Saad ED (2017) The impact of data errors on the outcome of randomized clinical trials. Clin Trials 14:499–506. 10.1177/1740774517716158 28641461 10.1177/1740774517716158

[96] Chmura Kraemer H, Periyakoil VS, Noda A (2002) Kappa coefficients in medical research. Stat Med 21:2109–2129 12111890 10.1002/sim.1180

[97] Landis JR, Koch GG (1977) The measurement of observer agreement for categorical data. Biometrics 33:159–174 843571

[98] Payton, Mark E (1996). Confidence intervals for the coefficient of variation. Conference on Applied Statistics in Agriculture. 10.4148/2475-7772.1320

[99] Gwet K (2021) Handbook of inter-rater reliability—the definitive guide to measuring the extent of agreement among raters. Advanced Analytics, LLC, Gaithersburg, USA

[100] Bakeman R, McArthur D, Quera V, Robinson BF (1997) Detecting sequential patterns and determining their reliability with fallible observers. Psychol Methods 2:357–370

