# Supplementary material for: Assessing the reliability of Response Evaluation Criteria In Solid Tumors (RECIST): a systematic review of the factors contributing to inter-observer variability
Source: Insights Imaging. 2026 Jun 30;17:180. doi: 10.1186/s13244-026-02320-6 (PMC13319625; doi:10.1186/s13244-026-02320-6)
Supplement: Supplementary file 1 — ELECTRONIC SUPPLEMENTARY MATERIAL [file 13244_2026_2320_MOESM1_ESM.pdf]

Assessing the reliability of Response Evaluation Criteria In Solid Tumors (RECIST): A systematic review of the factors contributing to inter-observer variability

ELECTRONIC SUPPLEMENTARY MATERIAL

1. Literature search

PubMed:

("Response Evaluation Criteria in Solid Tumors"[mesh] OR RECIST[tiab] OR "Response Evaluation Criteria in Solid Tumo*"[tiab]) AND (variabilit*[tiab] OR reproducibilit*[tiab]) AND 2008/12:2024[dp]

Embase.com:

('response evaluation criteria in solid tumors'/exp OR (RECIST OR 'Response Evaluation Criteria in Solid Tumo*'):ti,ab,kw) AND (variabilit* OR reproducibilit*):ti,ab,kw AND [2008-2024]/py

Scopus:

((TITLE-ABS(RECIST OR "Response Evaluation Criteria in Solid Tumo*") OR AUTHKEY(RECIST OR "Response Evaluation Criteria in Solid Tumo*")) AND (TITLE-ABS(variabilit* OR reproducibilit*) OR AUTHKEY(variabilit* OR reproducibilit*)) AND PUBYEAR > 2007
